# Supplementary material for: Investigating robust associations between functional connectivity based on graph theory and general intelligence
Source: Sci Rep. 2024 Jan 16;14:1368. doi: 10.1038/s41598-024-51333-y (PMC10791664; doi:10.1038/s41598-024-51333-y)
Supplement: Supplementary file 1 — Supplementary Information. [file 41598_2024_51333_MOESM1_ESM.pdf]

Supplementary Material

Investigating robust associations between functional connectivity based on graph theory and general intelligence

Dorothea Metzen, Christina Stammen, Christoph Fraenz, Caroline Schlüter, Wendy Johnson, Onur Güntürkün, Colin G. DeYoung, Erhan Genç

1. Intelligence measurement

A detailed description of all intelligence measures used for calculation of *g* can be found in Stammen et al. [1]. The following section will give a short overview of all used tests.

1.1. RUB Sample

Intelligence was assessed using the Intelligenz-Struktur-Test 2000 R (I-S-T 2000 R) [2], a well-established German intelligence test battery [2,3] that is largely comparable to the Wechsler Adult Intelligence Scale (WAIS) [4], the Bochumer Matrizentest (BOMAT) [5], a non-verbal German intelligence test that is comparable to the internationally established Raven's Advanced Progressive Matrices [6], the Bochumer Wissenstest (BOWIT) [7], a German questionnaire assessing general knowledge, and the Zahlenverbindungstest (ZVT) [8], a trail making test that measures cognitive processing speed.

1.2. HCP Sample

Intelligence was measured with the four subtests Penn Matrix Reasoning Task (PMAT) assessing reasoning ability, Short Penn Continuous Performance Test (SCPT) assessing visual attention, Variable Short Penn Line Orientation Test (VSPLOT) assessing visual-spatial processing, and Penn Word Memory Test (IWRD) assessing verbal episodic memory from the University of Pennsylvania Computerized Neurocognitive Battery (Penn CNB) [9,10] as well as the seven subtests Flanker Inhibitory Control and Attention Test (Flanker) assessing executive functions (attention), Dimensional Change Card Sort Test (CardSort) assessing executive functions (cognitive flexibility), List Sorting Working Memory Test (ListSort) assessing working memory capacity, Picture Sequence Memory Test (PicSeq) assessing episodic memory, Oral Reading Recognition Test (ReadEng) assessing reading decoding skills, Picture Vocabulary Test (PicVocab) assessing vocabulary knowledge, and Pattern Comparison Processing Speed Test (ProcSpeed) assessing processing speed from the NIH Toolbox for the Assessment of Neurological and Behavioral Function (http://www.nihtoolbox.org) [11,12].

1.3. UMN Sample

The UMN sample conducted the subtests Block Design (WAIS_BD) and Matrix Reasoning (WAIS_MR) to assess perceptual reasoning, Similarities (WAIS_SIM) and Vocabulary (WAIS_VC) to assess verbal comprehension, and Coding (WAIS_CD) to assess processing speed from the fourth edition Wechsler Adult Intelligence Scale (WAIS-IV) [13].

1.4. NKI Sample

Intelligence was measured using the four subtests Block Design (WASI_BD), Matrix Reasoning (WASI_MR), Similarities (WASI_SIM), and Vocabulary (WASI_VC) of the second edition Wechsler Abbreviated Scale of Intelligence (WASI-II) [14], which are conceptually comparable to the subtests from the WAIS-IV (see UMN Sample). However, WASI-II uses unique test items [15].

2. References

1 Stammen, C. *et al.* Robust associations between white matter microstructure and general intelligence. *Cereb Cortex.* **33**, 6723-6741 (2023).

2 Liepmann, D., Beauducel, A., Brocke, B. & Amthauer, R. Intelligenz-Struktur-Test 2000 R (I-S-T 2000 R). Manual., Vol. 2., erweiterte und überarbeitete Auflage (Hogrefe, 2007).

3 Beauducel, A., Brocke, B. & Liepmann, D. Perspectives on fluid and crystallized intelligence: facets for verbal, numerical, and figural intelligence. *Pers. Individ. Differ.* **30**, 977-994 (2001).

4 Erdodi, L. A. *et al.* Wechsler Adult Intelligence Scale-Fourth Edition (WAIS-IV) processing speed scores as measures of noncredible responding: The third generation of embedded performance validity indicators. *Psychol Assess.* **29**, 148-157 (2017).

5 Hossiep, R., Hasella, M. & Turck, D. BOMAT-advanced-short version: Bochumer Matrizentest. (Hogrefe, 2001).

6 Raven, J. C., Court, J. H. & Raven, J. Coloured progressive matrices. Manual for Raven's Progressive Matrices and Vocabulary Scales. (Oxford Psychologists Press, 1990).

7 Hossiep, R. & Schulte, M. BOWIT: Bochumer Wissenstest. (Hogrefe, 2008).

8 Oswald, W. D. & Roth, E. Der Zahlen-Verbindungs-Test (ZVT). (Hogrefe Verlag für Psychologie, 1987).

9 Gur, R. C. *et al.* Computerized neurocognitive scanning: I. Methodology and validation in healthy people. *NPP.* **25**, 766-776 (2001).

10 Gur, R. C. *et al.* A cognitive neuroscience-based computerized battery for efficient measurement of individual differences: standardization and initial construct validation. *J Neurosci Methods.* **187**, 254-262 (2010).

11 Gershon, R. C. *et al.* NIH Toolbox for the assessment of neurological and behavioral function. *Neurology.* **80**, S2-S6 (2013).

12 Weintraub, S. *et al.* Cognition assessment using the NIH Toolbox. *Neurology.* **80**, S54-S64 (2013).

13 Wechsler, D. Wechsler Adult Intelligence Scale - Forth edition (WAIS-IV). (Pearson Assessment, 2008).

14 Wechsler, D. Wechsler Abbreviated Intelligence Scale - Second edition (WASI-II). (NCS Pearson, 2011).
15 McCrimmon, A. W. & Smith, A. D. Review of the Wechsler Abbreviated Scale of Intelligence, Second Edition (WASI-II). *J Psychoeduc Assess.* **31**, 337-341 (2012).

Supplementary Table S1 List of all HCPMMP and subcortical areas with non-zero effect sizes for each data set (nodal efficiency). Left hemispheric areas are labeled "L", right hemispheric areas are labled "R". HCP sessions are depicted in blue, NKI sessions in orange,

HCP day 1		HCP day 2		HCP day 1 ses. 1		HCP day 1 ses. 2		HCP day 1& 2		NKI (TR = 645)		NKI (TR = 1400)		NKI (TR = 2500)		UMN		RUB	
Area	beta	Area	beta	Area	beta	Area	beta	Area	beta	Area	beta	Area	beta	Area	beta	Area	beta	Area	beta
L_7PC	-0,041	L_6ma	-0,036	L_55b	-0,058	L_a24	-0,005	L_6ma	-0,034	L_FEF	-0,030	L_V1	-0,053	L_V2	-0,029	L_a47r	0,038	L_POS2	-0,026
L_10v	-0,009	L_6mp	-0,003	L_RSC	-0,004	L_8Av	0,029	L_6mp	0,000	L_23c	-0,016	L_7PL	0,076	L_FEF	-0,060	L_9a	0,017	L_7Pm	-0,005
L_OFC	-0,004	L_a47r	0,023	L_MT	-0,030	L_LIPd	0,008	L_a47r	0,021	L_24dd	-0,028	L_45	0,026	L_PIT	-0,068	L_10v	0,026	L_5m	-0,002
L_STSda	0,001	L_PreS	-0,029	L_SFL	-0,015	L_TPOJ3	0,038	L_PreS	-0,023	L_8BL	0,008	L_47l	0,057	L_PCV	-0,026	L_13l	0,016	L_FST	-0,016
L_STSva	0,008	L_TPOJ1	-0,008	L_PCV	-0,031	L_PFm	0,003	L_TPOJ1	-0,005	L_45	0,003	L_a47r	0,018	L_7Pm	-0,052	R_V3B	0,040	R_LIPv	-0,021
R_V6	0,019	R_IFSp	-0,007	L_7m	0,013	L_31pd	0,007	R_IFSp	-0,003	L_6r	0,060	L_6r	0,108	L_23d	0,001	R_5L	0,021	R_25	0,016
R_10r	-0,003	R_11l	0,016	L_7PC	-0,058	L_PoI1	-0,013	R_11l	0,018	L_STSvp	0,010	L_a10p	0,006	L_v23ab	-0,046	R_a47r	0,038	R_p24	0,035
R_i6.8	0,000			L_6v	-0,012	R_V3B	0,019	R_IP2	0,002	L_VMV1	-0,026	L_13l	0,008	L_7AL	0,039	R_H	-0,002		
				L_a24	-0,020	R_31pv	0,016			L_pOFC	-0,002	L_OP2.3	-0,033	L_a24pr	0,008	R_FST	0,024		
				L_p32	-0,042	R_7AL	-0,027			R_V8	-0,026	L_EC	-0,003	L_8BM	0,025	R_hippo	-0,053		
				L_47m	0,004	R_p32	-0,003			R_8BL	0,007	L_PeEc	-0,028	L_p32	0,027				
				L_47l	0,001	R_10v	-0,012			R_10d	0,045	L_TE2a	-0,072	L_9m	0,010				
				L_IFJa	0,062	R_MI	-0,005			R_11l	0,018	L_TF	-0,018	L_8C	0,062				
				L_IFJp	0,020	R_Pir	0,000			R_Pir	-0,028	L_TE2p	-0,014	L_9.46d	-0,005				
				L_IFSa	0,005	R_AAIC	-0,019			R_H	-0,047	R_5m	-0,014	L_LIPd	-0,020				
				L_47s	0,036	R_31pd	0,018			R_31a	-0,061	R_5mv	-0,004	L_FOP1	0,013				
				L_LIPd	0,001	R_pOFC	-0,019			R_pOFC	-0,016	R_a24pr	0,001	L_H	-0,008				
				L_FOP2	-0,001	L_amy	-0,004			R_STSva	0,034	R_45	0,013	L_PHA1	-0,010				
				L_AIP	0,013	L_palli	-0,045			R_hippo	-0,005	R_a47r	0,031	L_TE2a	-0,069				
				L_PHA3	-0,002	R_acc	-0,027					R_PoI2	-0,026	L_DVT	-0,047				
				L_STSvp	0,002							R_FOP2	-0,014	L_PGp	0,016				
				L_TE1a	0,043							R_PreS	-0,086	L_IP0	0,058				
				L_PGs	0,008							R_STSvp	0,023	L_V4t	0,024				
				L_VMV3	-0,019							R_VMV2	-0,013	L_VMV2	-0,005				
				L_31pd	0,033							L_hippo	-0,009	L_VVC	-0,055				
				L_31a	0,002							R_cau	-0,008	L_Ig	-0,017				
				L_VVC	-0,004									L_TGv	-0,031				
				L_s32	-0,075									L_A4	0,060				
				L_TE1m	0,023									R_V1	-0,059				
				L_a32pr	-0,043									R_FFC	0,095				
				L_p24	-0,005									R_A1	-0,036				
				R_V6	0,083									R_7AL	0,004				
				R_RSC	-0,003									R_6ma	0,012				
				R_LO2	-0,017									R_7Am	0,069				

R_PCV	-0,005
R_7Pm	-0,006
R_6mp	-0,041
R_a24pr	-0,008
R_47m	-0,002
R_8C	0,002
R_13l	0,061
R_OFC	0,034
R_OP1	0,044
R_Pir	-0,030
R_PeEc	-0,002
R_STGa	0,012
R_TE1a	0,003
R_IP2	0,034
R_VMV3	-0,007
R_25	-0,033
R_pOFC	0,018
R_PoI1	0,008
R_FOP5	0,005
R_p10p	0,002
R_p47r	0,006
L_caud	-0,006
R_palli	-0,020

R_1	0,029
R_a24pr	0,078
R_10r	-0,002
R_8Ad	0,026
R_9m	0,009
R_IFJp	0,044
R_p9.46v	0,004
R_a10p	-0,073
R_11l	0,086
R_MI	-0,015
R_FOP3	0,035
R_EC	-0,062
R_PeEc	-0,021
R_PHA1	-0,031
R_STSda	-0,038
R_STSvp	0,043
R_TE1a	0,006
R_TE2p	-0,081
R_TPOJ2	-0,026
R_PFop	0,012
R_VMV1	-0,031
R_PHA2	0,010
R_25	-0,003
R_acc	-0,036
R_amy	0,028
R_puta	-0,012

Supplementary Table S2 List of all HCPMMP and subcortical areas overlapping between different datasets (nodal efficiency, see Figure 2). Left hemispheric areas are labeled "L", right hemispheric areas are labled

HCP across days		HCP same day		NKI		TR ≤ 720 ms data sets		TR = 2000 ms data sets	
**Area**	**beta**	**Area**	**beta**	**Area**	**beta**	**Area**	**beta**	**Area**	**beta**
-	-	L_a24	-0,013	-	-	R_11l	0,018	-	-
		L_LIPd	0,005						
		L_31pd	0,020						
		R_Pir	-0,015						
		R_pOFC	-0,001						

Supplementary Table S3 List of all HCPMMP and subcortical areas with non-zero effect sizes for each data set (local clustering). Left hemispheric areas are labeled "L", right hemispheric areas are labled "R". HCP sessions are depicted in blue, NKI sessions

HCP day 1		HCP day 2		HCP day 1 ses. 1		HCP day 1 ses. 2		HCP day 1 &2		NKI (TR = 645)		NKI (TR = 1400)		NKI (TR = 2500)		UMN		RUB	
Area	beta	Area	beta	Area	beta	Area	beta	Area	beta	Area	beta	Area	beta	Area	beta	Area	beta	Area	beta
L_5mv	-0,009	-	-	L_V7	0,012	-	-	-	-	L_PCV	0,029	L_PCV	0,032	L_44	0,010	-	-	-	-
L_23c	-0,009			L_FFC	-0,002					L_6d	0,036	L_VIP	0,024	L_IFJa	0,029				
L_MIP	0,021			L_LO2	-0,009					L_33pr	0,023	L_p32	0,004	L_IFJp	0,010				
L_33pr	-0,025			L_v23ab	0,012					L_45	0,032	L_44	0,025	L_RI	0,005				
L_8BL	0,004			L_7AL	-0,008					L_9a	-0,034	L_IFSp	0,008	L_TF	-0,050				
L_45	0,016			L_VIP	-0,012					R_MST	0,007	L_10v	-0,043	R_3b	0,010				
L_46	-0,012			L_6v	0,050					R_MIP	0,018	L_i6.8	0,043	R_6ma	0,038				
L_PeEc	0,018			L_47m	0,042					R_1	-0,020	L_s6.8	0,025	R_11l	0,009				
L_STSda	0,002			L_8C	-0,011					R_6mp	0,000	L_TE1a	0,051	R_s6.8	-0,036				
L_pOFC	-0,001			L_46	-0,010					R_a24	0,006	L_FST	-0,009	R_MI	0,047				
L_PoI1	0,018			L_a10p	0,030					R_p32	0,004	R_MST	0,052	R_FOP1	0,025				
R_3b	0,024			L_47s	0,001					R_IFJa	0,092	R_VIP	0,007	R_PreS	-0,022				
R_5m	0,016			L_PFcm	0,003					R_IFJp	0,041	R_33pr	0,003	R_TPOJ2	-0,004				
R_p32pr	-0,002			L_TF	0,015					R_11l	0,006	R_p32pr	0,041	L_caud	0,014				
R_9.46d	-0,034			L_PGp	-0,030					R_FOP1	0,015	R_47l	0,047	R_accu	-0,020				
R_9a	-0,045			L_PGi	0,025					R_EC	-0,006	R_a47r	0,016						
R_Pir	0,011			L_VMV1	0,004					R_H	-0,013	R_OFC	0,020						
R_AVI	0,021			L_s32	-0,016					R_FOP5	0,013	R_Pir	-0,064						
R_EC	-0,001			L_a32pr	-0,026					R_thala	-0,034	R_ProS	-0,041						
R_PBelt	0,002			R_V1	-0,031							R_STSda	0,012						
R_TPOJ2	-0,003			R_V3	0,039							R_IP0	-0,047						
R_VMV1	0,006			R_LO2	0,002							R_V4t	0,008						
R_V3CD	0,004			R_MT	-0,002							R_LO3	0,011						
R_p47r	0,013			R_5L	0,009														
R_caud	-0,001			R_a24pr	-0,019														
				R_p32	-0,018														
				R_10r	0,005														
				R_8C	0,034														
				R_a47r	0,003														

R_46	0,006
R_9a	-0,001
R_OP1	0,004
R_FOP2	0,001
R_STSvp	0,000
R_IP2	0,008
R_PGi	0,035
R_V4t	-0,008
R_V3CD	0,027
R_FOP5	0,001
R_STSva	0,001
L_amy	0,003
L_ventdc	0,002

Supplementary Table S4 Reliability in terms of ICC of all HCPMMP and subcortical areas (nodal efficiency & local clustering). Left hemispheric areas are labeled "L", right hemispheric areas are labeled "R".

Nodal Efficiency						Local Clustering					
HCP across days		HCP same day		NKI		HCP across days		HCP same day		NKI	
Area	ICC	Area	ICC	Area	ICC	Area	ICC	Area	ICC	Area	ICC
L_V1	0,372	L_V1	0,460	L_V1	0,281	L_V1	0,256	L_V1	0,376	L_V1	0,267
L_MST	0,391	L_MST	0,575	L_MST	0,293	L_MST	0,124	L_MST	0,267	L_MST	0,277
L_V6	0,403	L_V6	0,587	L_V6	0,316	L_V6	0,317	L_V6	0,297	L_V6	0,175
L_V2	0,434	L_V2	0,517	L_V2	0,332	L_V2	0,261	L_V2	0,251	L_V2	0,261
L_V3	0,415	L_V3	0,523	L_V3	0,278	L_V3	0,358	L_V3	0,280	L_V3	0,298
L_V4	0,390	L_V4	0,533	L_V4	0,306	L_V4	0,355	L_V4	0,354	L_V4	0,279
L_V8	0,395	L_V8	0,499	L_V8	0,263	L_V8	0,199	L_V8	0,174	L_V8	0,187
L_4	0,495	L_4	0,563	L_4	0,365	L_4	0,245	L_4	0,230	L_4	0,283
L_3b	0,473	L_3b	0,568	L_3b	0,312	L_3b	0,241	L_3b	0,216	L_3b	0,250
L_FEF	0,355	L_FEF	0,428	L_FEF	0,391	L_FEF	0,100	L_FEF	0,129	L_FEF	0,109
L_PEF	0,381	L_PEF	0,578	L_PEF	0,433	L_PEF	0,194	L_PEF	0,162	L_PEF	0,112
L_55b	0,446	L_55b	0,496	L_55b	0,378	L_55b	0,194	L_55b	0,191	L_55b	0,126
L_V3A	0,399	L_V3A	0,529	L_V3A	0,287	L_V3A	0,398	L_V3A	0,368	L_V3A	0,222
L_RSC	0,292	L_RSC	0,308	L_RSC	0,225	L_RSC	0,110	L_RSC	0,085	L_RSC	0,171
L_POS2	0,334	L_POS2	0,487	L_POS2	0,270	L_POS2	0,222	L_POS2	0,110	L_POS2	0,181
L_V7	0,397	L_V7	0,527	L_V7	0,370	L_V7	0,286	L_V7	0,224	L_V7	0,159
L_IPS1	0,387	L_IPS1	0,540	L_IPS1	0,335	L_IPS1	0,200	L_IPS1	0,298	L_IPS1	0,224
L_FFC	0,400	L_FFC	0,481	L_FFC	0,346	L_FFC	0,194	L_FFC	0,171	L_FFC	0,204
L_V3B	0,419	L_V3B	0,538	L_V3B	0,324	L_V3B	0,254	L_V3B	0,211	L_V3B	0,167
L_LO1	0,373	L_LO1	0,493	L_LO1	0,314	L_LO1	0,374	L_LO1	0,367	L_LO1	0,267
L_LO2	0,353	L_LO2	0,408	L_LO2	0,387	L_LO2	0,240	L_LO2	0,257	L_LO2	0,241
L_PIT	0,366	L_PIT	0,508	L_PIT	0,349	L_PIT	0,204	L_PIT	0,224	L_PIT	0,182
L_MT	0,360	L_MT	0,551	L_MT	0,341	L_MT	0,112	L_MT	0,264	L_MT	0,302
L_A1	0,335	L_A1	0,505	L_A1	0,419	L_A1	0,085	L_A1	0,162	L_A1	0,300

L_PSL	0,431	L_PSL	0,499	L_PSL	0,376
L_SFL	0,374	L_SFL	0,496	L_SFL	0,306
L_PCV	0,278	L_PCV	0,451	L_PCV	0,313
L_STV	0,456	L_STV	0,523	L_STV	0,340
L_7Pm	0,352	L_7Pm	0,488	L_7Pm	0,265
L_7m	0,314	L_7m	0,419	L_7m	0,250
L_POS1	0,312	L_POS1	0,418	L_POS1	0,224
L_23d	0,278	L_23d	0,261	L_23d	0,292
L_v23ab	0,300	L_v23ab	0,402	L_v23ab	0,270
L_d23ab	0,283	L_d23ab	0,379	L_d23ab	0,283
L_31pv	0,250	L_31pv	0,438	L_31pv	0,280
L_5m	0,399	L_5m	0,509	L_5m	0,415
L_5mv	0,318	L_5mv	0,500	L_5mv	0,426
L_23c	0,286	L_23c	0,436	L_23c	0,359
L_5L	0,405	L_5L	0,462	L_5L	0,278
L_24dd	0,340	L_24dd	0,443	L_24dd	0,398
L_24dv	0,330	L_24dv	0,408	L_24dv	0,420
L_7AL	0,374	L_7AL	0,465	L_7AL	0,269
L_SCEF	0,332	L_SCEF	0,515	L_SCEF	0,396
L_6ma	0,351	L_6ma	0,453	L_6ma	0,365
L_7Am	0,346	L_7Am	0,428	L_7Am	0,276
L_7PL	0,398	L_7PL	0,482	L_7PL	0,232
L_7PC	0,363	L_7PC	0,511	L_7PC	0,332
L_LIPv	0,389	L_LIPv	0,517	L_LIPv	0,325
L_VIP	0,386	L_VIP	0,446	L_VIP	0,278
L_MIP	0,347	L_MIP	0,569	L_MIP	0,285
L_1	0,437	L_1	0,538	L_1	0,286
L_2	0,422	L_2	0,539	L_2	0,341
L_3a	0,550	L_3a	0,625	L_3a	0,340
L_6d	0,398	L_6d	0,530	L_6d	0,311

L_PSL	0,225	L_PSL	0,234	L_PSL	0,189
L_SFL	0,151	L_SFL	0,266	L_SFL	0,133
L_PCV	0,108	L_PCV	0,136	L_PCV	0,193
L_STV	0,220	L_STV	0,162	L_STV	0,236
L_7Pm	0,178	L_7Pm	0,182	L_7Pm	0,108
L_7m	0,206	L_7m	0,262	L_7m	0,224
L_POS1	0,114	L_POS1	0,109	L_POS1	0,147
L_23d	0,118	L_23d	-0,002	L_23d	0,194
L_v23ab	0,175	L_v23ab	0,182	L_v23ab	0,136
L_d23ab	0,140	L_d23ab	0,160	L_d23ab	0,176
L_31pv	0,130	L_31pv	0,243	L_31pv	0,175
L_5m	0,244	L_5m	0,242	L_5m	0,212
L_5mv	0,176	L_5mv	0,173	L_5mv	0,152
L_23c	0,164	L_23c	0,073	L_23c	0,184
L_5L	0,153	L_5L	0,119	L_5L	0,139
L_24dd	0,103	L_24dd	0,101	L_24dd	0,235
L_24dv	0,114	L_24dv	0,059	L_24dv	0,154
L_7AL	0,232	L_7AL	0,194	L_7AL	0,096
L_SCEF	0,182	L_SCEF	0,138	L_SCEF	0,119
L_6ma	0,059	L_6ma	0,135	L_6ma	0,067
L_7Am	0,267	L_7Am	0,217	L_7Am	0,161
L_7PL	0,269	L_7PL	0,306	L_7PL	0,179
L_7PC	0,210	L_7PC	0,200	L_7PC	0,103
L_LIPv	0,179	L_LIPv	0,273	L_LIPv	0,148
L_VIP	0,197	L_VIP	0,291	L_VIP	0,162
L_MIP	0,225	L_MIP	0,225	L_MIP	0,207
L_1	0,225	L_1	0,334	L_1	0,233
L_2	0,200	L_2	0,153	L_2	0,176
L_3a	0,254	L_3a	0,216	L_3a	0,246
L_6d	0,230	L_6d	0,274	L_6d	0,208

L_6mp	0,358	L_6mp	0,483	L_6mp	0,375
L_6v	0,340	L_6v	0,422	L_6v	0,369
L_p24pr	0,350	L_p24pr	0,282	L_p24pr	0,420
L_33pr	0,304	L_33pr	0,199	L_33pr	0,318
L_a24pr	0,291	L_a24pr	0,260	L_a24pr	0,404
L_p32pr	0,273	L_p32pr	0,370	L_p32pr	0,439
L_a24	0,243	L_a24	0,346	L_a24	0,315
L_d32	0,301	L_d32	0,439	L_d32	0,336
L_8BM	0,373	L_8BM	0,454	L_8BM	0,364
L_p32	0,255	L_p32	0,330	L_p32	0,282
L_10r	0,292	L_10r	0,319	L_10r	0,286
L_47m	0,408	L_47m	0,455	L_47m	0,271
L_8Av	0,335	L_8Av	0,456	L_8Av	0,322
L_8Ad	0,324	L_8Ad	0,449	L_8Ad	0,325
L_9m	0,320	L_9m	0,429	L_9m	0,292
L_8BL	0,400	L_8BL	0,485	L_8BL	0,361
L_9p	0,379	L_9p	0,433	L_9p	0,329
L_10d	0,336	L_10d	0,375	L_10d	0,313
L_8C	0,401	L_8C	0,533	L_8C	0,341
L_44	0,423	L_44	0,527	L_44	0,402
L_45	0,428	L_45	0,510	L_45	0,346
L_47l	0,462	L_47l	0,557	L_47l	0,342
L_a47r	0,345	L_a47r	0,343	L_a47r	0,243
L_6r	0,396	L_6r	0,540	L_6r	0,465
L_IFJa	0,355	L_IFJa	0,499	L_IFJa	0,405
L_IFJp	0,346	L_IFJp	0,523	L_IFJp	0,413
L_IFSp	0,388	L_IFSp	0,549	L_IFSp	0,347
L_IFSa	0,353	L_IFSa	0,488	L_IFSa	0,380
L_p9.46v	0,352	L_p9.46v	0,449	L_p9.46v	0,311
L_46	0,386	L_46	0,378	L_46	0,306

L_6mp	0,153	L_6mp	0,204	L_6mp	0,232
L_6v	0,106	L_6v	0,181	L_6v	0,150
L_p24pr	0,173	L_p24pr	0,007	L_p24pr	0,153
L_33pr	0,182	L_33pr	0,059	L_33pr	0,181
L_a24pr	0,149	L_a24pr	0,031	L_a24pr	0,244
L_p32pr	0,152	L_p32pr	0,070	L_p32pr	0,245
L_a24	0,117	L_a24	0,110	L_a24	0,101
L_d32	0,140	L_d32	0,200	L_d32	0,145
L_8BM	0,144	L_8BM	0,201	L_8BM	0,151
L_p32	0,106	L_p32	0,140	L_p32	0,145
L_10r	0,089	L_10r	0,071	L_10r	0,165
L_47m	0,212	L_47m	0,144	L_47m	0,076
L_8Av	0,198	L_8Av	0,315	L_8Av	0,141
L_8Ad	0,124	L_8Ad	0,201	L_8Ad	0,160
L_9m	0,164	L_9m	0,165	L_9m	0,101
L_8BL	0,174	L_8BL	0,229	L_8BL	0,195
L_9p	0,215	L_9p	0,201	L_9p	0,128
L_10d	0,097	L_10d	0,217	L_10d	0,085
L_8C	0,159	L_8C	0,289	L_8C	0,158
L_44	0,219	L_44	0,281	L_44	0,100
L_45	0,171	L_45	0,267	L_45	0,165
L_47l	0,164	L_47l	0,211	L_47l	0,157
L_a47r	0,130	L_a47r	0,097	L_a47r	0,114
L_6r	0,115	L_6r	0,203	L_6r	0,139
L_IFJa	0,125	L_IFJa	0,117	L_IFJa	0,129
L_IFJp	0,186	L_IFJp	0,190	L_IFJp	0,228
L_IFSp	0,207	L_IFSp	0,208	L_IFSp	0,125
L_IFSa	0,105	L_IFSa	0,243	L_IFSa	0,156
L_p9.46v	0,141	L_p9.46v	0,143	L_p9.46v	0,187
L_46	0,166	L_46	0,227	L_46	0,121

L_a9.46v	0,353	L_a9.46v	0,413	L_a9.46v	0,210
L_9.46d	0,433	L_9.46d	0,396	L_9.46d	0,329
L_9a	0,409	L_9a	0,423	L_9a	0,325
L_10v	0,361	L_10v	0,273	L_10v	0,285
L_a10p	0,310	L_a10p	0,287	L_a10p	0,303
L_10pp	0,399	L_10pp	0,256	L_10pp	0,233
L_11l	0,377	L_11l	0,235	L_11l	0,179
L_13l	0,483	L_13l	0,238	L_13l	0,273
L_OFC	0,461	L_OFC	0,194	L_OFC	0,230
L_47s	0,465	L_47s	0,480	L_47s	0,325
L_LIPd	0,300	L_LIPd	0,535	L_LIPd	0,318
L_6a	0,289	L_6a	0,379	L_6a	0,315
L_i6.8	0,361	L_i6.8	0,508	L_i6.8	0,314
L_s6.8	0,395	L_s6.8	0,435	L_s6.8	0,335
L_43	0,428	L_43	0,543	L_43	0,378
L_OP4	0,473	L_OP4	0,545	L_OP4	0,355
L_OP1	0,455	L_OP1	0,563	L_OP1	0,383
L_OP2.3	0,375	L_OP2.3	0,505	L_OP2.3	0,435
L_52	0,296	L_52	0,431	L_52	0,342
L_RI	0,376	L_RI	0,511	L_RI	0,391
L_PFcm	0,433	L_PFcm	0,535	L_PFcm	0,413
L_PoI2	0,313	L_PoI2	0,406	L_PoI2	0,432
L_TA2	0,371	L_TA2	0,431	L_TA2	0,384
L_FOP4	0,366	L_FOP4	0,503	L_FOP4	0,454
L_MI	0,420	L_MI	0,400	L_MI	0,442
L_Pir	0,458	L_Pir	0,262	L_Pir	0,286
L_AVI	0,448	L_AVI	0,374	L_AVI	0,389
L_AAIC	0,501	L_AAIC	0,277	L_AAIC	0,426
L_FOP1	0,407	L_FOP1	0,525	L_FOP1	0,473
L_FOP3	0,266	L_FOP3	0,269	L_FOP3	0,513

L_a9.46v	0,087	L_a9.46v	0,136	L_a9.46v	0,057
L_9.46d	0,117	L_9.46d	0,153	L_9.46d	0,197
L_9a	0,227	L_9a	0,286	L_9a	0,181
L_10v	0,186	L_10v	0,052	L_10v	0,047
L_a10p	0,187	L_a10p	0,075	L_a10p	0,147
L_10pp	0,133	L_10pp	0,080	L_10pp	0,126
L_11l	0,154	L_11l	0,074	L_11l	0,108
L_13l	0,163	L_13l	0,057	L_13l	0,132
L_OFC	0,180	L_OFC	0,018	L_OFC	0,071
L_47s	0,203	L_47s	0,146	L_47s	0,134
L_LIPd	0,155	L_LIPd	0,278	L_LIPd	0,220
L_6a	0,104	L_6a	0,097	L_6a	0,122
L_i6.8	0,224	L_i6.8	0,214	L_i6.8	0,160
L_s6.8	0,167	L_s6.8	0,264	L_s6.8	0,129
L_43	0,179	L_43	0,170	L_43	0,199
L_OP4	0,246	L_OP4	0,170	L_OP4	0,208
L_OP1	0,099	L_OP1	0,180	L_OP1	0,230
L_OP2.3	0,122	L_OP2.3	0,161	L_OP2.3	0,241
L_52	0,095	L_52	0,087	L_52	0,203
L_RI	0,153	L_RI	0,061	L_RI	0,255
L_PFcm	0,185	L_PFcm	0,135	L_PFcm	0,152
L_PoI2	0,156	L_PoI2	0,067	L_PoI2	0,172
L_TA2	0,183	L_TA2	0,152	L_TA2	0,253
L_FOP4	0,179	L_FOP4	0,123	L_FOP4	0,254
L_MI	0,137	L_MI	0,094	L_MI	0,226
L_Pir	0,294	L_Pir	0,076	L_Pir	0,021
L_AVI	0,278	L_AVI	0,083	L_AVI	0,140
L_AAIC	0,307	L_AAIC	0,033	L_AAIC	0,163
L_FOP1	0,157	L_FOP1	0,150	L_FOP1	0,184
L_FOP3	0,151	L_FOP3	0,092	L_FOP3	0,268

L_FOP2	0,292	L_FOP2	0,375	L_FOP2	0,512
L_PFt	0,350	L_PFt	0,518	L_PFt	0,355
L_AIP	0,335	L_AIP	0,485	L_AIP	0,340
L_EC	0,357	L_EC	0,239	L_EC	0,248
L_PreS	0,317	L_PreS	0,371	L_PreS	0,327
L_H	0,333	L_H	0,276	L_H	0,342
L_ProS	0,414	L_ProS	0,514	L_ProS	0,326
L_PeEc	0,498	L_PeEc	0,244	L_PeEc	0,291
L_STGa	0,413	L_STGa	0,362	L_STGa	0,443
L_PBelt	0,473	L_PBelt	0,586	L_PBelt	0,410
L_A5	0,440	L_A5	0,554	L_A5	0,413
L_PHA1	0,327	L_PHA1	0,329	L_PHA1	0,349
L_PHA3	0,397	L_PHA3	0,301	L_PHA3	0,363
L_STSda	0,366	L_STSda	0,515	L_STSda	0,391
L_STSdp	0,359	L_STSdp	0,497	L_STSdp	0,377
L_STSvp	0,403	L_STSvp	0,573	L_STSvp	0,368
L_TGd	0,443	L_TGd	0,255	L_TGd	0,293
L_TE1a	0,432	L_TE1a	0,304	L_TE1a	0,336
L_TE1p	0,382	L_TE1p	0,440	L_TE1p	0,265
L_TE2a	0,488	L_TE2a	0,324	L_TE2a	0,312
L_TF	0,498	L_TF	0,239	L_TF	0,385
L_TE2p	0,455	L_TE2p	0,358	L_TE2p	0,415
L_PHT	0,461	L_PHT	0,402	L_PHT	0,302
L_PH	0,369	L_PH	0,503	L_PH	0,327
L_TPOJ1	0,432	L_TPOJ1	0,568	L_TPOJ1	0,367
L_TPOJ2	0,408	L_TPOJ2	0,583	L_TPOJ2	0,292
L_TPOJ3	0,404	L_TPOJ3	0,613	L_TPOJ3	0,381
L_DVT	0,404	L_DVT	0,541	L_DVT	0,247
L_PGp	0,320	L_PGp	0,404	L_PGp	0,280
L_IP2	0,360	L_IP2	0,510	L_IP2	0,328

L_FOP2	0,127	L_FOP2	0,112	L_FOP2	0,252
L_PFt	0,160	L_PFt	0,237	L_PFt	0,208
L_AIP	0,180	L_AIP	0,126	L_AIP	0,078
L_EC	0,194	L_EC	0,025	L_EC	0,049
L_PreS	0,097	L_PreS	0,104	L_PreS	0,204
L_H	0,201	L_H	0,139	L_H	0,136
L_ProS	0,200	L_ProS	0,279	L_ProS	0,238
L_PeEc	0,170	L_PeEc	0,029	L_PeEc	0,110
L_STGa	0,102	L_STGa	0,055	L_STGa	0,224
L_PBelt	0,163	L_PBelt	0,222	L_PBelt	0,346
L_A5	0,112	L_A5	0,079	L_A5	0,246
L_PHA1	0,117	L_PHA1	0,060	L_PHA1	0,176
L_PHA3	0,214	L_PHA3	0,076	L_PHA3	0,195
L_STSda	0,226	L_STSda	0,120	L_STSda	0,342
L_STSdp	0,127	L_STSdp	0,181	L_STSdp	0,258
L_STSvp	0,146	L_STSvp	0,272	L_STSvp	0,173
L_TGd	0,178	L_TGd	0,043	L_TGd	0,110
L_TE1a	0,134	L_TE1a	0,076	L_TE1a	0,102
L_TE1p	0,123	L_TE1p	0,164	L_TE1p	0,169
L_TE2a	0,209	L_TE2a	0,088	L_TE2a	0,137
L_TF	0,141	L_TF	0,027	L_TF	0,067
L_TE2p	0,097	L_TE2p	0,059	L_TE2p	0,121
L_PHT	0,064	L_PHT	0,224	L_PHT	0,159
L_PH	0,186	L_PH	0,258	L_PH	0,164
L_TPOJ1	0,183	L_TPOJ1	0,126	L_TPOJ1	0,171
L_TPOJ2	0,177	L_TPOJ2	0,229	L_TPOJ2	0,225
L_TPOJ3	0,166	L_TPOJ3	0,202	L_TPOJ3	0,126
L_DVT	0,223	L_DVT	0,245	L_DVT	0,176
L_PGp	0,216	L_PGp	0,205	L_PGp	0,243
L_IP2	0,157	L_IP2	0,178	L_IP2	0,132

L_IP1	0,360	L_IP1	0,539	L_IP1	0,292
L_IP0	0,330	L_IP0	0,494	L_IP0	0,278
L_PFop	0,437	L_PFop	0,535	L_PFop	0,361
L_PF	0,343	L_PF	0,477	L_PF	0,342
L_PFm	0,376	L_PFm	0,443	L_PFm	0,252
L_PGi	0,439	L_PGi	0,557	L_PGi	0,304
L_PGs	0,437	L_PGs	0,440	L_PGs	0,271
L_V6A	0,369	L_V6A	0,503	L_V6A	0,325
L_VMV1	0,336	L_VMV1	0,453	L_VMV1	0,329
L_VMV3	0,353	L_VMV3	0,422	L_VMV3	0,292
L_PHA2	0,338	L_PHA2	0,302	L_PHA2	0,361
L_V4t	0,415	L_V4t	0,550	L_V4t	0,354
L_FST	0,440	L_FST	0,520	L_FST	0,295
L_V3CD	0,389	L_V3CD	0,526	L_V3CD	0,300
L_LO3	0,363	L_LO3	0,460	L_LO3	0,359
L_VMV2	0,324	L_VMV2	0,411	L_VMV2	0,380
L_31pd	0,315	L_31pd	0,442	L_31pd	0,328
L_31a	0,241	L_31a	0,364	L_31a	0,318
L_VVC	0,355	L_VVC	0,449	L_VVC	0,301
L_25	0,447	L_25	0,178	L_25	0,316
L_s32	0,469	L_s32	0,277	L_s32	0,308
L_pOFC	0,438	L_pOFC	0,138	L_pOFC	0,302
L_PoI1	0,293	L_PoI1	0,307	L_PoI1	0,351
L_Ig	0,303	L_Ig	0,470	L_Ig	0,432
L_FOP5	0,413	L_FOP5	0,393	L_FOP5	0,396
L_p10p	0,338	L_p10p	0,310	L_p10p	0,253
L_p47r	0,396	L_p47r	0,451	L_p47r	0,348
L_TGv	0,436	L_TGv	0,257	L_TGv	0,333
L_MBelt	0,386	L_MBelt	0,512	L_MBelt	0,446
L_LBelt	0,435	L_LBelt	0,617	L_LBelt	0,402

L_IP1	0,164	L_IP1	0,206	L_IP1	0,200
L_IP0	0,206	L_IP0	0,205	L_IP0	0,197
L_PFop	0,186	L_PFop	0,180	L_PFop	0,199
L_PF	0,183	L_PF	0,306	L_PF	0,173
L_PFm	0,172	L_PFm	0,245	L_PFm	0,161
L_PGi	0,236	L_PGi	0,320	L_PGi	0,195
L_PGs	0,228	L_PGs	0,212	L_PGs	0,094
L_V6A	0,214	L_V6A	0,260	L_V6A	0,133
L_VMV1	0,131	L_VMV1	0,201	L_VMV1	0,173
L_VMV3	0,156	L_VMV3	0,145	L_VMV3	0,226
L_PHA2	0,238	L_PHA2	0,066	L_PHA2	0,149
L_V4t	0,196	L_V4t	0,252	L_V4t	0,187
L_FST	0,215	L_FST	0,252	L_FST	0,216
L_V3CD	0,326	L_V3CD	0,324	L_V3CD	0,216
L_LO3	0,201	L_LO3	0,269	L_LO3	0,230
L_VMV2	0,128	L_VMV2	0,155	L_VMV2	0,215
L_31pd	0,201	L_31pd	0,189	L_31pd	0,211
L_31a	0,114	L_31a	0,094	L_31a	0,182
L_VVC	0,131	L_VVC	0,099	L_VVC	0,277
L_25	0,260	L_25	0,031	L_25	0,128
L_s32	0,274	L_s32	0,043	L_s32	0,183
L_pOFC	0,245	L_pOFC	-0,011	L_pOFC	0,136
L_PoI1	0,153	L_PoI1	0,098	L_PoI1	0,097
L_Ig	0,124	L_Ig	0,127	L_Ig	0,261
L_FOP5	0,341	L_FOP5	0,141	L_FOP5	0,200
L_p10p	0,134	L_p10p	0,055	L_p10p	0,162
L_p47r	0,117	L_p47r	0,178	L_p47r	0,153
L_TGv	0,153	L_TGv	0,020	L_TGv	0,107
L_MBelt	0,118	L_MBelt	0,163	L_MBelt	0,306
L_LBelt	0,190	L_LBelt	0,200	L_LBelt	0,369

L_A4	0,461	L_A4	0,514	L_A4	0,377
L_STSva	0,444	L_STSva	0,504	L_STSva	0,392
L_TE1m	0,428	L_TE1m	0,421	L_TE1m	0,315
L_PI	0,358	L_PI	0,312	L_PI	0,187
L_a32pr	0,307	L_a32pr	0,436	L_a32pr	0,385
L_p24	0,257	L_p24	0,271	L_p24	0,356
R_V1	0,390	R_V1	0,470	R_V1	0,297
R_MST	0,444	R_MST	0,511	R_MST	0,356
R_V6	0,395	R_V6	0,583	R_V6	0,285
R_V2	0,401	R_V2	0,551	R_V2	0,319
R_V3	0,397	R_V3	0,519	R_V3	0,274
R_V4	0,386	R_V4	0,503	R_V4	0,293
R_V8	0,369	R_V8	0,501	R_V8	0,324
R_4	0,454	R_4	0,586	R_4	0,366
R_3b	0,470	R_3b	0,561	R_3b	0,336
R_FEF	0,461	R_FEF	0,477	R_FEF	0,386
R_PEF	0,344	R_PEF	0,563	R_PEF	0,374
R_55b	0,479	R_55b	0,536	R_55b	0,404
R_V3A	0,393	R_V3A	0,535	R_V3A	0,268
R_RSC	0,249	R_RSC	0,369	R_RSC	0,251
R_POS2	0,380	R_POS2	0,510	R_POS2	0,301
R_V7	0,387	R_V7	0,531	R_V7	0,309
R_IPS1	0,347	R_IPS1	0,526	R_IPS1	0,320
R_FFC	0,352	R_FFC	0,421	R_FFC	0,309
R_V3B	0,343	R_V3B	0,512	R_V3B	0,323
R_LO1	0,385	R_LO1	0,503	R_LO1	0,304
R_LO2	0,299	R_LO2	0,348	R_LO2	0,327
R_PIT	0,254	R_PIT	0,340	R_PIT	0,379
R_MT	0,455	R_MT	0,529	R_MT	0,366
R_A1	0,352	R_A1	0,483	R_A1	0,424

L_A4	0,147	L_A4	0,147	L_A4	0,282
L_STSva	0,155	L_STSva	0,233	L_STSva	0,244
L_TE1m	0,209	L_TE1m	0,067	L_TE1m	0,098
L_PI	0,213	L_PI	0,118	L_PI	0,054
L_a32pr	0,162	L_a32pr	0,142	L_a32pr	0,164
L_p24	0,080	L_p24	0,081	L_p24	0,132
R_V1	0,324	R_V1	0,257	R_V1	0,291
R_MST	0,190	R_MST	0,234	R_MST	0,356
R_V6	0,216	R_V6	0,254	R_V6	0,221
R_V2	0,306	R_V2	0,218	R_V2	0,238
R_V3	0,420	R_V3	0,373	R_V3	0,257
R_V4	0,362	R_V4	0,409	R_V4	0,252
R_V8	0,140	R_V8	0,154	R_V8	0,186
R_4	0,261	R_4	0,249	R_4	0,279
R_3b	0,214	R_3b	0,278	R_3b	0,280
R_FEF	0,221	R_FEF	0,149	R_FEF	0,152
R_PEF	0,142	R_PEF	0,217	R_PEF	0,167
R_55b	0,172	R_55b	0,172	R_55b	0,104
R_V3A	0,381	R_V3A	0,359	R_V3A	0,225
R_RSC	0,053	R_RSC	0,027	R_RSC	0,155
R_POS2	0,217	R_POS2	0,181	R_POS2	0,175
R_V7	0,268	R_V7	0,261	R_V7	0,170
R_IPS1	0,203	R_IPS1	0,245	R_IPS1	0,193
R_FFC	0,127	R_FFC	0,117	R_FFC	0,136
R_V3B	0,269	R_V3B	0,313	R_V3B	0,212
R_LO1	0,263	R_LO1	0,237	R_LO1	0,245
R_LO2	0,249	R_LO2	0,262	R_LO2	0,241
R_PIT	0,190	R_PIT	0,212	R_PIT	0,231
R_MT	0,207	R_MT	0,233	R_MT	0,289
R_A1	0,140	R_A1	0,133	R_A1	0,277

R_PSL	0,370	R_PSL	0,475	R_PSL	0,348
R_SFL	0,341	R_SFL	0,466	R_SFL	0,350
R_PCV	0,325	R_PCV	0,470	R_PCV	0,359
R_STV	0,373	R_STV	0,537	R_STV	0,333
R_7Pm	0,325	R_7Pm	0,457	R_7Pm	0,282
R_7m	0,271	R_7m	0,393	R_7m	0,252
R_POS1	0,316	R_POS1	0,434	R_POS1	0,235
R_23d	0,195	R_23d	0,209	R_23d	0,277
R_v23ab	0,304	R_v23ab	0,371	R_v23ab	0,312
R_d23ab	0,269	R_d23ab	0,288	R_d23ab	0,317
R_31pv	0,275	R_31pv	0,442	R_31pv	0,361
R_5m	0,403	R_5m	0,532	R_5m	0,382
R_5mv	0,324	R_5mv	0,497	R_5mv	0,389
R_23c	0,276	R_23c	0,436	R_23c	0,358
R_5L	0,408	R_5L	0,479	R_5L	0,319
R_24dd	0,352	R_24dd	0,498	R_24dd	0,406
R_24dv	0,322	R_24dv	0,383	R_24dv	0,441
R_7AL	0,393	R_7AL	0,504	R_7AL	0,242
R_SCEF	0,311	R_SCEF	0,436	R_SCEF	0,405
R_6ma	0,362	R_6ma	0,389	R_6ma	0,410
R_7Am	0,375	R_7Am	0,466	R_7Am	0,279
R_7PL	0,331	R_7PL	0,514	R_7PL	0,267
R_7PC	0,397	R_7PC	0,481	R_7PC	0,294
R_LIPv	0,383	R_LIPv	0,548	R_LIPv	0,283
R_VIP	0,406	R_VIP	0,483	R_VIP	0,274
R_MIP	0,357	R_MIP	0,528	R_MIP	0,268
R_1	0,513	R_1	0,551	R_1	0,311
R_2	0,414	R_2	0,577	R_2	0,308
R_3a	0,478	R_3a	0,628	R_3a	0,370
R_6d	0,474	R_6d	0,501	R_6d	0,310

R_PSL	0,167	R_PSL	0,217	R_PSL	0,166
R_SFL	0,141	R_SFL	0,152	R_SFL	0,106
R_PCV	0,147	R_PCV	0,108	R_PCV	0,177
R_STV	0,155	R_STV	0,104	R_STV	0,209
R_7Pm	0,189	R_7Pm	0,226	R_7Pm	0,108
R_7m	0,188	R_7m	0,163	R_7m	0,285
R_POS1	0,140	R_POS1	0,176	R_POS1	0,056
R_23d	0,064	R_23d	-0,004	R_23d	0,133
R_v23ab	0,126	R_v23ab	0,175	R_v23ab	0,193
R_d23ab	0,127	R_d23ab	0,074	R_d23ab	0,146
R_31pv	0,122	R_31pv	0,175	R_31pv	0,178
R_5m	0,202	R_5m	0,266	R_5m	0,242
R_5mv	0,097	R_5mv	0,117	R_5mv	0,120
R_23c	0,156	R_23c	0,102	R_23c	0,167
R_5L	0,208	R_5L	0,193	R_5L	0,090
R_24dd	0,123	R_24dd	0,102	R_24dd	0,174
R_24dv	0,085	R_24dv	0,025	R_24dv	0,231
R_7AL	0,135	R_7AL	0,219	R_7AL	0,114
R_SCEF	0,105	R_SCEF	0,070	R_SCEF	0,196
R_6ma	0,162	R_6ma	0,173	R_6ma	0,217
R_7Am	0,229	R_7Am	0,225	R_7Am	0,159
R_7PL	0,182	R_7PL	0,250	R_7PL	0,156
R_7PC	0,238	R_7PC	0,279	R_7PC	0,119
R_LIPv	0,174	R_LIPv	0,247	R_LIPv	0,143
R_VIP	0,255	R_VIP	0,243	R_VIP	0,155
R_MIP	0,214	R_MIP	0,240	R_MIP	0,217
R_1	0,270	R_1	0,366	R_1	0,189
R_2	0,180	R_2	0,211	R_2	0,237
R_3a	0,236	R_3a	0,177	R_3a	0,152
R_6d	0,252	R_6d	0,284	R_6d	0,297

R_6mp	0,370	R_6mp	0,461	R_6mp	0,392
R_6v	0,483	R_6v	0,533	R_6v	0,355
R_p24pr	0,295	R_p24pr	0,299	R_p24pr	0,366
R_33pr	0,295	R_33pr	0,255	R_33pr	0,330
R_a24pr	0,218	R_a24pr	0,221	R_a24pr	0,380
R_p32pr	0,230	R_p32pr	0,413	R_p32pr	0,412
R_a24	0,364	R_a24	0,314	R_a24	0,315
R_d32	0,365	R_d32	0,404	R_d32	0,373
R_8BM	0,261	R_8BM	0,437	R_8BM	0,396
R_p32	0,379	R_p32	0,290	R_p32	0,315
R_10r	0,296	R_10r	0,254	R_10r	0,299
R_47m	0,438	R_47m	0,394	R_47m	0,258
R_8Av	0,472	R_8Av	0,502	R_8Av	0,334
R_8Ad	0,290	R_8Ad	0,406	R_8Ad	0,352
R_9m	0,361	R_9m	0,393	R_9m	0,260
R_8BL	0,334	R_8BL	0,518	R_8BL	0,350
R_9p	0,365	R_9p	0,444	R_9p	0,345
R_10d	0,301	R_10d	0,350	R_10d	0,336
R_8C	0,404	R_8C	0,504	R_8C	0,361
R_44	0,404	R_44	0,499	R_44	0,430
R_45	0,416	R_45	0,502	R_45	0,390
R_47l	0,402	R_47l	0,423	R_47l	0,355
R_a47r	0,331	R_a47r	0,309	R_a47r	0,253
R_6r	0,407	R_6r	0,531	R_6r	0,406
R_IFJa	0,319	R_IFJa	0,513	R_IFJa	0,377
R_IFJp	0,342	R_IFJp	0,465	R_IFJp	0,380
R_IFSp	0,395	R_IFSp	0,564	R_IFSp	0,335
R_IFSa	0,445	R_IFSa	0,534	R_IFSa	0,347
R_p9.46v	0,393	R_p9.46v	0,518	R_p9.46v	0,321
R_46	0,317	R_46	0,456	R_46	0,284

R_6mp	0,153	R_6mp	0,245	R_6mp	0,220
R_6v	0,117	R_6v	0,211	R_6v	0,179
R_p24pr	0,110	R_p24pr	0,074	R_p24pr	0,118
R_33pr	0,101	R_33pr	0,053	R_33pr	0,225
R_a24pr	0,038	R_a24pr	0,022	R_a24pr	0,234
R_p32pr	0,103	R_p32pr	0,122	R_p32pr	0,234
R_a24	0,205	R_a24	0,068	R_a24	0,166
R_d32	0,185	R_d32	0,125	R_d32	0,102
R_8BM	0,099	R_8BM	0,164	R_8BM	0,113
R_p32	0,196	R_p32	0,050	R_p32	0,187
R_10r	0,135	R_10r	-0,003	R_10r	0,085
R_47m	0,217	R_47m	0,118	R_47m	0,058
R_8Av	0,197	R_8Av	0,287	R_8Av	0,147
R_8Ad	0,149	R_8Ad	0,175	R_8Ad	0,121
R_9m	0,169	R_9m	0,174	R_9m	0,139
R_8BL	0,155	R_8BL	0,251	R_8BL	0,146
R_9p	0,222	R_9p	0,262	R_9p	0,126
R_10d	0,052	R_10d	0,180	R_10d	0,188
R_8C	0,173	R_8C	0,301	R_8C	0,112
R_44	0,170	R_44	0,115	R_44	0,209
R_45	0,119	R_45	0,096	R_45	0,126
R_47l	0,181	R_47l	0,147	R_47l	0,093
R_a47r	0,152	R_a47r	0,115	R_a47r	0,108
R_6r	0,188	R_6r	0,193	R_6r	0,224
R_IFJa	0,076	R_IFJa	0,145	R_IFJa	0,164
R_IFJp	0,093	R_IFJp	0,097	R_IFJp	0,237
R_IFSp	0,173	R_IFSp	0,209	R_IFSp	0,154
R_IFSa	0,075	R_IFSa	0,220	R_IFSa	0,091
R_p9.46v	0,253	R_p9.46v	0,293	R_p9.46v	0,164
R_46	0,180	R_46	0,241	R_46	0,100

R_a9.46v	0,324	R_a9.46v	0,372	R_a9.46v	0,276
R_9.46d	0,347	R_9.46d	0,429	R_9.46d	0,266
R_9a	0,337	R_9a	0,429	R_9a	0,305
R_10v	0,377	R_10v	0,294	R_10v	0,323
R_a10p	0,320	R_a10p	0,231	R_a10p	0,341
R_10pp	0,436	R_10pp	0,256	R_10pp	0,287
R_11l	0,377	R_11l	0,267	R_11l	0,214
R_13l	0,461	R_13l	0,227	R_13l	0,296
R_OFC	0,418	R_OFC	0,259	R_OFC	0,277
R_47s	0,507	R_47s	0,390	R_47s	0,351
R_LIPd	0,357	R_LIPd	0,515	R_LIPd	0,314
R_6a	0,341	R_6a	0,408	R_6a	0,333
R_i6.8	0,443	R_i6.8	0,431	R_i6.8	0,335
R_s6.8	0,347	R_s6.8	0,477	R_s6.8	0,359
R_43	0,396	R_43	0,535	R_43	0,405
R_OP4	0,400	R_OP4	0,573	R_OP4	0,410
R_OP1	0,416	R_OP1	0,587	R_OP1	0,414
R_OP2.3	0,408	R_OP2.3	0,513	R_OP2.3	0,396
R_52	0,311	R_52	0,456	R_52	0,388
R_RI	0,387	R_RI	0,554	R_RI	0,443
R_PFcm	0,371	R_PFcm	0,543	R_PFcm	0,398
R_PoI2	0,351	R_PoI2	0,451	R_PoI2	0,429
R_TA2	0,301	R_TA2	0,436	R_TA2	0,401
R_FOP4	0,357	R_FOP4	0,464	R_FOP4	0,449
R_MI	0,319	R_MI	0,352	R_MI	0,441
R_Pir	0,387	R_Pir	0,309	R_Pir	0,277
R_AVI	0,425	R_AVI	0,433	R_AVI	0,351
R_AAIC	0,413	R_AAIC	0,380	R_AAIC	0,408
R_FOP1	0,382	R_FOP1	0,479	R_FOP1	0,486
R_FOP3	0,311	R_FOP3	0,460	R_FOP3	0,458

R_a9.46v	0,143	R_a9.46v	0,148	R_a9.46v	0,079
R_9.46d	0,223	R_9.46d	0,183	R_9.46d	0,173
R_9a	0,129	R_9a	0,210	R_9a	0,165
R_10v	0,122	R_10v	0,038	R_10v	0,089
R_a10p	0,148	R_a10p	0,094	R_a10p	0,150
R_10pp	0,164	R_10pp	0,078	R_10pp	0,170
R_11l	0,136	R_11l	0,113	R_11l	0,084
R_13l	0,136	R_13l	0,090	R_13l	0,091
R_OFC	0,197	R_OFC	0,066	R_OFC	0,162
R_47s	0,161	R_47s	0,061	R_47s	0,125
R_LIPd	0,132	R_LIPd	0,196	R_LIPd	0,148
R_6a	0,114	R_6a	0,098	R_6a	0,135
R_i6.8	0,071	R_i6.8	0,147	R_i6.8	0,132
R_s6.8	0,134	R_s6.8	0,170	R_s6.8	0,134
R_43	0,201	R_43	0,075	R_43	0,227
R_OP4	0,277	R_OP4	0,278	R_OP4	0,181
R_OP1	0,170	R_OP1	0,172	R_OP1	0,266
R_OP2.3	0,113	R_OP2.3	0,200	R_OP2.3	0,289
R_52	0,123	R_52	0,126	R_52	0,293
R_RI	0,155	R_RI	0,168	R_RI	0,238
R_PFcm	0,136	R_PFcm	0,203	R_PFcm	0,230
R_PoI2	0,076	R_PoI2	0,079	R_PoI2	0,198
R_TA2	0,036	R_TA2	0,086	R_TA2	0,251
R_FOP4	0,236	R_FOP4	0,145	R_FOP4	0,270
R_MI	0,174	R_MI	0,049	R_MI	0,255
R_Pir	0,306	R_Pir	0,105	R_Pir	0,034
R_AVI	0,246	R_AVI	0,071	R_AVI	0,166
R_AAIC	0,166	R_AAIC	0,104	R_AAIC	0,139
R_FOP1	0,124	R_FOP1	0,108	R_FOP1	0,183
R_FOP3	0,153	R_FOP3	0,126	R_FOP3	0,253

R_FOP2	0,313	R_FOP2	0,466	R_FOP2	0,488
R_PFt	0,415	R_PFt	0,555	R_PFt	0,338
R_AIP	0,405	R_AIP	0,555	R_AIP	0,285
R_EC	0,369	R_EC	0,225	R_EC	0,258
R_PreS	0,392	R_PreS	0,383	R_PreS	0,304
R_H	0,000	R_H	0,000	R_H	0,340
R_ProS	0,413	R_ProS	0,539	R_ProS	0,332
R_PeEc	0,500	R_PeEc	0,157	R_PeEc	0,302
R_STGa	0,330	R_STGa	0,414	R_STGa	0,409
R_PBelt	0,451	R_PBelt	0,585	R_PBelt	0,447
R_A5	0,431	R_A5	0,583	R_A5	0,381
R_PHA1	0,289	R_PHA1	0,319	R_PHA1	0,386
R_PHA3	0,372	R_PHA3	0,238	R_PHA3	0,351
R_STSda	0,384	R_STSda	0,466	R_STSda	0,419
R_STSdp	0,380	R_STSdp	0,533	R_STSdp	0,428
R_STSvp	0,398	R_STSvp	0,539	R_STSvp	0,337
R_TGd	0,466	R_TGd	0,293	R_TGd	0,323
R_TE1a	0,359	R_TE1a	0,355	R_TE1a	0,358
R_TE1p	0,312	R_TE1p	0,359	R_TE1p	0,323
R_TE2a	0,504	R_TE2a	0,301	R_TE2a	0,368
R_TF	0,452	R_TF	0,307	R_TF	0,378
R_TE2p	0,429	R_TE2p	0,233	R_TE2p	0,416
R_PHT	0,386	R_PHT	0,425	R_PHT	0,321
R_PH	0,354	R_PH	0,458	R_PH	0,347
R_TPOJ1	0,434	R_TPOJ1	0,543	R_TPOJ1	0,348
R_TPOJ2	0,437	R_TPOJ2	0,569	R_TPOJ2	0,285
R_TPOJ3	0,376	R_TPOJ3	0,488	R_TPOJ3	0,310
R_DVT	0,389	R_DVT	0,501	R_DVT	0,235
R_PGp	0,336	R_PGp	0,441	R_PGp	0,284
R_IP2	0,450	R_IP2	0,534	R_IP2	0,334

R_FOP2	0,082	R_FOP2	0,166	R_FOP2	0,229
R_PFt	0,223	R_PFt	0,223	R_PFt	0,207
R_AIP	0,276	R_AIP	0,214	R_AIP	0,234
R_EC	0,184	R_EC	-0,001	R_EC	0,016
R_PreS	0,112	R_PreS	0,068	R_PreS	0,146
R_H	0,000	R_H	0,000	R_H	0,269
R_ProS	0,130	R_ProS	0,190	R_ProS	0,203
R_PeEc	0,119	R_PeEc	0,003	R_PeEc	0,076
R_STGa	0,144	R_STGa	0,053	R_STGa	0,194
R_PBelt	0,177	R_PBelt	0,185	R_PBelt	0,305
R_A5	0,101	R_A5	0,178	R_A5	0,238
R_PHA1	0,073	R_PHA1	0,085	R_PHA1	0,229
R_PHA3	0,208	R_PHA3	0,099	R_PHA3	0,169
R_STSda	0,142	R_STSda	0,035	R_STSda	0,173
R_STSdp	0,144	R_STSdp	0,099	R_STSdp	0,223
R_STSvp	0,136	R_STSvp	0,139	R_STSvp	0,158
R_TGd	0,249	R_TGd	0,002	R_TGd	0,166
R_TE1a	0,113	R_TE1a	0,084	R_TE1a	0,118
R_TE1p	0,092	R_TE1p	0,086	R_TE1p	0,179
R_TE2a	0,165	R_TE2a	0,109	R_TE2a	0,086
R_TF	0,193	R_TF	0,004	R_TF	0,116
R_TE2p	0,162	R_TE2p	-0,022	R_TE2p	0,167
R_PHT	0,124	R_PHT	0,174	R_PHT	0,115
R_PH	0,169	R_PH	0,203	R_PH	0,154
R_TPOJ1	0,154	R_TPOJ1	0,156	R_TPOJ1	0,212
R_TPOJ2	0,197	R_TPOJ2	0,220	R_TPOJ2	0,212
R_TPOJ3	0,100	R_TPOJ3	0,176	R_TPOJ3	0,161
R_DVT	0,250	R_DVT	0,219	R_DVT	0,242
R_PGp	0,170	R_PGp	0,213	R_PGp	0,216
R_IP2	0,237	R_IP2	0,239	R_IP2	0,187

R_IP1	0,358	R_IP1	0,507	R_IP1	0,269
R_IP0	0,343	R_IP0	0,465	R_IP0	0,279
R_PFop	0,401	R_PFop	0,576	R_PFop	0,346
R_PF	0,483	R_PF	0,503	R_PF	0,344
R_PFm	0,435	R_PFm	0,472	R_PFm	0,265
R_PGi	0,376	R_PGi	0,520	R_PGi	0,263
R_PGs	0,331	R_PGs	0,480	R_PGs	0,269
R_V6A	0,358	R_V6A	0,461	R_V6A	0,309
R_VMV1	0,300	R_VMV1	0,471	R_VMV1	0,347
R_VMV3	0,360	R_VMV3	0,448	R_VMV3	0,299
R_PHA2	0,344	R_PHA2	0,264	R_PHA2	0,345
R_V4t	0,436	R_V4t	0,525	R_V4t	0,386
R_FST	0,412	R_FST	0,535	R_FST	0,348
R_V3CD	0,407	R_V3CD	0,526	R_V3CD	0,329
R_LO3	0,396	R_LO3	0,491	R_LO3	0,320
R_VMV2	0,360	R_VMV2	0,492	R_VMV2	0,357
R_31pd	0,273	R_31pd	0,457	R_31pd	0,389
R_31a	0,220	R_31a	0,304	R_31a	0,352
R_VVC	0,338	R_VVC	0,441	R_VVC	0,306
R_25	0,443	R_25	0,176	R_25	0,307
R_s32	0,441	R_s32	0,270	R_s32	0,322
R_pOFC	0,433	R_pOFC	0,199	R_pOFC	0,359
R_PoI1	0,326	R_PoI1	0,409	R_PoI1	0,441
R_Ig	0,387	R_Ig	0,440	R_Ig	0,444
R_FOP5	0,456	R_FOP5	0,479	R_FOP5	0,389
R_p10p	0,310	R_p10p	0,305	R_p10p	0,240
R_p47r	0,391	R_p47r	0,400	R_p47r	0,290
R_TGv	0,518	R_TGv	0,199	R_TGv	0,323
R_MBelt	0,371	R_MBelt	0,506	R_MBelt	0,460
R_LBelt	0,418	R_LBelt	0,587	R_LBelt	0,435

R_IP1	0,280	R_IP1	0,279	R_IP1	0,185
R_IP0	0,185	R_IP0	0,236	R_IP0	0,157
R_PFop	0,217	R_PFop	0,226	R_PFop	0,214
R_PF	0,255	R_PF	0,281	R_PF	0,289
R_PFm	0,244	R_PFm	0,291	R_PFm	0,175
R_PGi	0,144	R_PGi	0,321	R_PGi	0,176
R_PGs	0,226	R_PGs	0,306	R_PGs	0,125
R_V6A	0,257	R_V6A	0,177	R_V6A	0,135
R_VMV1	0,209	R_VMV1	0,206	R_VMV1	0,245
R_VMV3	0,145	R_VMV3	0,186	R_VMV3	0,278
R_PHA2	0,246	R_PHA2	0,063	R_PHA2	0,161
R_V4t	0,162	R_V4t	0,204	R_V4t	0,190
R_FST	0,192	R_FST	0,180	R_FST	0,216
R_V3CD	0,265	R_V3CD	0,297	R_V3CD	0,201
R_LO3	0,225	R_LO3	0,271	R_LO3	0,174
R_VMV2	0,193	R_VMV2	0,139	R_VMV2	0,247
R_31pd	0,212	R_31pd	0,249	R_31pd	0,122
R_31a	0,081	R_31a	0,109	R_31a	0,151
R_VVC	0,165	R_VVC	0,163	R_VVC	0,202
R_25	0,294	R_25	0,069	R_25	0,128
R_s32	0,279	R_s32	0,057	R_s32	0,168
R_pOFC	0,201	R_pOFC	0,096	R_pOFC	0,111
R_PoI1	0,104	R_PoI1	0,027	R_PoI1	0,193
R_Ig	0,099	R_Ig	0,161	R_Ig	0,299
R_FOP5	0,321	R_FOP5	0,126	R_FOP5	0,253
R_p10p	0,119	R_p10p	0,101	R_p10p	0,144
R_p47r	0,162	R_p47r	0,219	R_p47r	0,138
R_TGv	0,154	R_TGv	0,099	R_TGv	0,132
R_MBelt	0,092	R_MBelt	0,158	R_MBelt	0,325
R_LBelt	0,157	R_LBelt	0,195	R_LBelt	0,326

R_A4	0,382	R_A4	0,538	R_A4	0,418
R_STSva	0,438	R_STSva	0,470	R_STSva	0,377
R_TE1m	0,439	R_TE1m	0,338	R_TE1m	0,307
R_PI	0,279	R_PI	0,292	R_PI	0,287
R_a32pr	0,341	R_a32pr	0,379	R_a32pr	0,407
R_p24	0,362	R_p24	0,283	R_p24	0,371
L_accumbens	0,471	L_accumbens	0,264	L_accumbens.a	0,312
L_amygdala	0,408	L_amygdala	0,321	L_amygdala	0,193
L_caudate	0,306	L_caudate	0,399	L_caudate	0,355
L_hippocampu	0,320	L_hippocampu	0,390	L_hippocampu	0,194
L_pallidum	0,231	L_pallidum	0,241	L_pallidum	0,355
L_putamen	0,258	L_putamen	0,381	L_putamen	0,357
L_thalamus	0,203	L_thalamus	0,299	L_thalamus	0,233
L_ventraldc	0,247	L_ventraldc	0,333	L_ventraldc	0,186
R_accumbens	0,517	R_accumbens	0,226	R_accumbens	0,279
R_amygdala	0,360	R_amygdala	0,329	R_amygdala	0,292
R_caudate	0,334	R_caudate	0,345	R_caudate	0,364
R_hippocampu	0,304	R_hippocampu	0,355	R_hippocampu	0,226
R_pallidum	0,210	R_pallidum	0,251	R_pallidum	0,344
R_putamen	0,292	R_putamen	0,344	R_putamen	0,367
R_thalamus	0,233	R_thalamus	0,308	R_thalamus	0,280
R_ventraldc	0,174	R_ventraldc	0,304	R_ventraldc	0,264

R_A4	0,104	R_A4	0,124	R_A4	0,260
R_STSva	0,170	R_STSva	0,104	R_STSva	0,214
R_TE1m	0,194	R_TE1m	0,058	R_TE1m	0,181
R_PI	0,149	R_PI	0,079	R_PI	0,084
R_a32pr	0,213	R_a32pr	0,077	R_a32pr	0,186
R_p24	0,194	R_p24	0,102	R_p24	0,176
L_accumbens	0,272	L_accumbens	0,152	L_accumbens.a	0,095
L_amygdala	0,244	L_amygdala	0,088	L_amygdala	0,035
L_caudate	0,126	L_caudate	0,040	L_caudate	0,091
L_hippocampu	0,189	L_hippocampu	0,032	L_hippocampu	0,021
L_pallidum	0,213	L_pallidum	0,037	L_pallidum	0,111
L_putamen	0,099	L_putamen	0,037	L_putamen	0,178
L_thalamus	0,009	L_thalamus	0,030	L_thalamus_pr	0,109
L_ventraldc	0,040	L_ventraldc	0,142	L_ventraldc	0,051
R_accumbens	0,244	R_accumbens	0,114	R_accumbens.a	0,176
R_amygdala	0,254	R_amygdala	0,028	R_amygdala	0,003
R_caudate	0,155	R_caudate	0,071	R_caudate	0,048
R_hippocampu	0,088	R_hippocampu	0,029	R_hippocampu	0,094
R_pallidum	0,202	R_pallidum	0,024	R_pallidum	0,138
R_putamen	0,078	R_putamen	0,089	R_putamen	0,156
R_thalamus	0,126	R_thalamus	0,107	R_thalamus_pr	0,115
R_ventraldc	0,060	R_ventraldc	0,038	R_ventraldc	-0,006

Supplementary Table S5 Results of multiple regression analyses for nodal efficiency. The areas with non-zero effect sizes identified via elastic-net in one sample (left column) have been used as predictors for general intelligence in all other samples. Depicted are the coefficients of determination R^2 and the respective p-value (uncorrected for multiple comparisons). Control variables were age, sex, age*sex, age², age²*sex, handedness, total brain volume, and head motion.

	**predictors**	**HCP day 1**		**HCP day 2**		**HCP day 1 ses. 1**		**HCP day 1 ses. 2**		**HCP day 1&2**		**NKI (TR=645)**		**NKI (TR=1400)**		**NKI (TR=2500)**		**UMN**		**RUB**	
		R^2	*p*	R^2	*p*	R^2	*p*	R^2	*p*	R^2	*p*	R^2	*p*	R^2	*p*	R^2	*p*	R^2	*p*	R^2	*p*
**HCP day 1**	8	.05	<.001	.01	.249	.02	.001	.01	.273	.01	.313	.02	.331	.01	.872	.02	.456	.04	.286	.01	.739
**HCP day 2**	7	.00	.651	.04	<.001	.01	.110	.01	.033	.04	<.001	.02	.298	.05	.013	.03	.082	.08	.002	.01	.714
**HCP day 1 ses. 1**	57	.08	.013	.08	.025	.21	<.001	.10	<.001	.08	.033	.21	.011	.23	.012	.16	.263	.17	.856	.12	.162
**HCP day 1 ses. 2**	20	.04	.002	.02	.435	.04	.003	.08	<.001	.02	.459	.10	.009	.05	.678	.04	.753	.11	.052	.03	.587
**HCP day 1& 2**	8	.01	.0393	.05	<.001	.02	.017	.02	.014	.05	<.001	.03	.126	.05	.023	.04	.034	.09	.001	.01	.677
**NKI (TR = 645)**	19	.02	.556	.02	.268	.03	.035	.03	.045	.02	.295	.18	<.001	.13	<.001	.08	.151	.11	.035	.04	.345
**NKI (TR = 1400)**	26	.02	.535	.03	.200	.02	.700	.02	.610	.03	.234	.07	.336	.30	<.001	.11	.025	.16	.010	.04	.665
**NKI (TR = 2500)**	60	.07	.051	.06	.601	.06	.249	.07	.097	.05	.662	.18	.203	.27	<.001	.46	<.001	.24	.283	.11	.462
**UMN**	10	.02	.022	.01	.171	.00	.881	.02	.004	.01	.191	.06	.014	.07	.009	.02	.740	.18	<.001	.01	.764
**RUB**	7	.00	.856	.01	.044	.01	.131	.01	.216	.01	.067	.02	.430	.04	.034	.03	.122	.05	.057	.07	<.001

Supplementary Table S6 Results of multiple regression analyses for nodal efficiency. The areas with non-zero effect sizes identified via elastic-net in one sample (left column) have been used as predictors for general intelligence in all other samples. Depicted is the number of significant predictors in the regression analyses along with its percentage compared to the number of predictors identified via elastic-net (uncorrected for multiple comparisons). Control variables were age, sex, age*sex, age², age²*sex, handedness, total brain volume, and head motion.

	predictors	HCP day 1	HCP day 2	HCP day 1 ses. 1	HCP day 1 ses. 2	HCP day 1&2	NKI (TR=645)	NKI (TR=1400)	NKI (TR=2500)	UMN	RUB
**HCP day 1**	8	4 (50%)	1 (13%)	2 (25%)	0 (0%)	1 (13%)	1 (13%)	0 (0%)	0 (0%)	1 (13%)	0 (0%)
**HCP day 2**	7	0 (0%)	2 (29%)	0 (0%)	2 (29%)	2 (29%)	0 (0%)	2 (29%)	0 (0%)	2 (29%)	0 (0%)
**HCP day 1 ses. 1**	57	4 (7%)	6 (11%)	15 (26%)	11 (19%)	6 (11%)	4 (7%)	7 (12%)	2 (4%)	1 (2%)	6 (11%)
**HCP day 1 ses. 2**	20	2 (10%)	0 (0%)	4 (20%)	4 (20%)	1 (5%)	3 (15%)	0 (0%)	0 (0%)	2 (10%)	0 (0%)
**HCP day 1&2**	8	0 (0%)	5 (63%)	2 (25%)	3 (38%)	5 (63%)	1 (13%)	2 (25%)	2 (25%)	1 (13%)	0 (0%)
**NKI (TR = 645)**	19	1 (5%)	2 (11%)	3 (16%)	2 (11%)	2 (11%)	5 (26%)	2 (11%)	4 (21%)	1 (5%)	1 (5%)
**NKI (TR = 1400)**	26	1 (4%)	1 (4%)	1 (4%)	1 (4%)	1 (4%)	0 (0%)	10 (38%)	3 (12%)	4 (15%)	0 (0%)
**NKI (TR = 2500)**	60	9 (15%)	4 (7%)	3 (5%)	5 (8%)	4 (7%)	4 (7%)	5 (8%)	20 (33%)	3 (5%)	4 (7%)
**UMN**	10	2 (20%)	1 (10%)	0 (0%)	3 (30%)	1 (10%)	3 (30%)	0 (0%)	0 (0%)	2 (20%)	0 (0%)
**RUB**	7	0 (0%)	1 (14%)	0 (0%)	0 (0%)	1 (14%)	0 (0%)	1 (14%)	2 (29%)	2 (29%)	2 (29%)

Supplementary Table S7 Results of multiple regression analyses for local clustering. The areas with non-zero effect sizes identified via elastic-net in one sample (left column) have been used as predictors for general intelligence in all other samples. Depicted are the coefficients of determination R^2 and the respective p-value (uncorrected for multiple comparisons). Control variables were age, sex, age*sex, age², age²*sex, handedness, total brain volume, and head motion.

		**HCP day 1**		**HCP day 2**		**HCP day 1 ses. 1**		**HCP day 1 ses. 2**		**HCP day 1&2**		**NKI (TR=645)**		**NKI (TR=1400)**		**NKI (TR=2500)**		**UMN**		**RUB**	
	**predictors**	R^2	*p*	R^2	*p*	R^2	*p*	R^2	*p*	R^2	*p*	R^2	*p*	R^2	*p*	R^2	*p*	R^2	*p*	R^2	*p*
**HCP day 1**	25	.13	<.001	.04	.068	.03	.310	.03	.110	.04	.069	.08	.153	.11	.037	.08	.271	.08	.636	.03	.803
**HCP day 2**	0	-	-	-	-	-	-	-	-	-	-	-	-	-	-	-	-	-	-	-	-
**HCP day 1 ses. 1**	42	.07	.005	.04	.698	.18	<.001	.06	.038	.04	.698	.09	.843	.13	.434	.12	.340	.18	.214	.10	.112
**HCP day 1 ses. 2**	0	-	-	-	-	-	-	-	-	-	-	-	-	-	-	-	-	-	-	-	-
**HCP day 1&2**	0	-	-	-	-	-	-	-	-	-	-	-	-	-	-	-	-	-	-	-	-
**NKI (TR = 645)**	19	.03	.042	.01	.984	.02	.368	.02	.247	.01	.979	.23	<.001	.09	.053	.07	.108	.09	.128	.01	>.999
**NKI (TR = 1400)**	23	.03	.194	.02	.603	.03	.109	.02	.312	.02	.579	.08	.085	.29	<.001	.09	.083	.09	.382	.05	.331
**NKI (TR = 2500)**	15	.02	.240	.02	.150	.02	.357	.02	.285	.02	.126	.06	.098	.04	.430	.20	<.001	.07	.271	.04	.140
**UMN**	0	-	-	-	-	-	-	-	-	-	-	-	-	-	-	-	-	-	-	-	-
**RUB**	0	-	-	-	-	-	-	-	-	-	-	-	-	-	-	-	-	-	-	-	-

Supplementary Table S8 Results of multiple regression analyses for local clustering. The areas with non-zero effect sizes identified via elastic-net in one sample (left column) have been used as predictors for general intelligence in all other samples. Depicted is the number of significant predictors in the regression analyses along with its percentage compared to the number of predictors identified via elastic-net (uncorrected for multiple comparisons). Control variables were age, sex, age*sex, age², age²*sex, handedness, total brain volume, and head motion.

	predictors	HCP day 1	HCP day 2	HCP day 1 ses. 1	HCP day 1 ses. 2	HCP day 1&2	NKI (TR=645)	NKI (TR=1400)	NKI (TR=2500)	UMN	RUB
**HCP day 1**	25	17 (68%)	3 (12%)	1 (4%)	1 (4%)	3 (12%)	2 (8%)	3 (12%)	2 (8%)	1 (4%)	0 (0%)
**HCP day 2**	0	-	-	-	-	-	-	-	-	-	-
**HCP day 1 ses. 1**	42	4 (10%)	2 (5%)	24 (57%)	5 (12%)	2 (5%)	2 (5%)	1 (2%)	2 (5%)	5 (12%)	3 (7%)
**HCP day 1 ses. 2**	0	-	-	-	-	-	-	-	-	-	-
**HCP day 1&2**	0	-	-	-	-	-	-	-	-	-	-
**NKI (TR = 645)**	19	2 (11%)	0 (0%)	1 (5%)	1 (5%)	0 (0%)	8 (42%)	2 (11%)	3 (16%)	2 (11%)	0 (0%)
**NKI (TR = 1400)**	23	2 (9%)	2 (9%)	0 (0%)	0 (0%)	2 (9%)	3 (13%)	10 (43%)	2 (9%)	2 (9%)	2 (9%)
**NKI (TR = 2500)**	15	2 (13%)	2 (13%)	0 (0%)	1 (7%)	2 (13%)	1 (7%)	1 (7%)	7 (47%)	2 (13%)	2 (13%)
**UMN**	0	-	-	-	-	-	-	-	-	-	-
**RUB**	0	-	-	-	-	-	-	-	-	-	-

Supplementary Table S9 Split-half reliability of global graph metrics.

	Global efficiency	Global clustering	Small-world propensity
**HCP day 1 session 1**	0,99	0,97	0,97
**NKI (TR = 645)**	0,99	0,99	0,98
**NKI (TR = 1400)**	0,98	0,96	0,96
**NKI (TR = 2500)**	0,96	0,92	0,97
**UMN**	0,95	0,90	0,93
**RUB**	0,96	0,92	0,96

Supplementary Table S10 Split-half reliability of all HCPMMP and subcortical areas (nodal efficiency). Left hemispheric areas are labeled "L", right hemispheric areas are labeled "R".

HCP day 1 session 1		NKI (TR = 645)		NKI (TR = 1400)		NKI (TR = 2500)		UMN		RUB	
Area	SB	Area	SB	Area	SB	Area	SB	Area	SB	Area	SB
L_V1	0,990	L_V1	0,984	L_V1	0,961	L_V1	0,930	L_V1	0,887	L_V1	0,916
L_MST	0,985	L_MST	0,987	L_MST	0,964	L_MST	0,929	L_MST	0,893	L_MST	0,930
L_V6	0,990	L_V6	0,984	L_V6	0,959	L_V6	0,910	L_V6	0,900	L_V6	0,923
L_V2	0,990	L_V2	0,985	L_V2	0,965	L_V2	0,919	L_V2	0,905	L_V2	0,940
L_V3	0,991	L_V3	0,985	L_V3	0,970	L_V3	0,922	L_V3	0,914	L_V3	0,939
L_V4	0,991	L_V4	0,986	L_V4	0,974	L_V4	0,923	L_V4	0,911	L_V4	0,941
L_V8	0,989	L_V8	0,983	L_V8	0,966	L_V8	0,920	L_V8	0,877	L_V8	0,926
L_4	0,993	L_4	0,988	L_4	0,973	L_4	0,941	L_4	0,925	L_4	0,950
L_3b	0,992	L_3b	0,990	L_3b	0,971	L_3b	0,931	L_3b	0,920	L_3b	0,945
L_FEF	0,982	L_FEF	0,979	L_FEF	0,965	L_FEF	0,922	L_FEF	0,911	L_FEF	0,924
L_PEF	0,986	L_PEF	0,982	L_PEF	0,966	L_PEF	0,923	L_PEF	0,914	L_PEF	0,937
L_55b	0,990	L_55b	0,980	L_55b	0,965	L_55b	0,916	L_55b	0,925	L_55b	0,932
L_V3A	0,991	L_V3A	0,985	L_V3A	0,968	L_V3A	0,917	L_V3A	0,913	L_V3A	0,941
L_RSC	0,979	L_RSC	0,985	L_RSC	0,954	L_RSC	0,896	L_RSC	0,891	L_RSC	0,904
L_POS2	0,987	L_POS2	0,983	L_POS2	0,965	L_POS2	0,906	L_POS2	0,908	L_POS2	0,924
L_V7	0,989	L_V7	0,990	L_V7	0,969	L_V7	0,922	L_V7	0,909	L_V7	0,933
L_IPS1	0,987	L_IPS1	0,990	L_IPS1	0,966	L_IPS1	0,913	L_IPS1	0,904	L_IPS1	0,938
L_FFC	0,989	L_FFC	0,987	L_FFC	0,962	L_FFC	0,921	L_FFC	0,911	L_FFC	0,928
L_V3B	0,986	L_V3B	0,986	L_V3B	0,968	L_V3B	0,902	L_V3B	0,905	L_V3B	0,931
L_LO1	0,988	L_LO1	0,985	L_LO1	0,971	L_LO1	0,915	L_LO1	0,907	L_LO1	0,926
L_LO2	0,987	L_LO2	0,988	L_LO2	0,967	L_LO2	0,923	L_LO2	0,921	L_LO2	0,927
L_PIT	0,989	L_PIT	0,987	L_PIT	0,967	L_PIT	0,917	L_PIT	0,909	L_PIT	0,919
L_MT	0,987	L_MT	0,989	L_MT	0,970	L_MT	0,926	L_MT	0,910	L_MT	0,937
L_A1	0,982	L_A1	0,986	L_A1	0,970	L_A1	0,937	L_A1	0,906	L_A1	0,939
L_PSL	0,988	L_PSL	0,984	L_PSL	0,967	L_PSL	0,931	L_PSL	0,894	L_PSL	0,929

L_SFL	0,988	L_SFL	0,981	L_SFL	0,957	L_SFL	0,912	L_SFL	0,918	L_SFL	0,937
L_PCV	0,985	L_PCV	0,987	L_PCV	0,966	L_PCV	0,908	L_PCV	0,902	L_PCV	0,921
L_STV	0,988	L_STV	0,985	L_STV	0,972	L_STV	0,928	L_STV	0,897	L_STV	0,936
L_7Pm	0,989	L_7Pm	0,989	L_7Pm	0,963	L_7Pm	0,902	L_7Pm	0,923	L_7Pm	0,929
L_7m	0,982	L_7m	0,985	L_7m	0,969	L_7m	0,900	L_7m	0,896	L_7m	0,938
L_POS1	0,984	L_POS1	0,984	L_POS1	0,943	L_POS1	0,903	L_POS1	0,899	L_POS1	0,925
L_23d	0,977	L_23d	0,978	L_23d	0,963	L_23d	0,892	L_23d	0,917	L_23d	0,913
L_v23ab	0,981	L_v23ab	0,984	L_v23ab	0,948	L_v23ab	0,900	L_v23ab	0,905	L_v23ab	0,925
L_d23ab	0,981	L_d23ab	0,983	L_d23ab	0,952	L_d23ab	0,906	L_d23ab	0,887	L_d23ab	0,916
L_31pv	0,980	L_31pv	0,981	L_31pv	0,955	L_31pv	0,897	L_31pv	0,895	L_31pv	0,914
L_5m	0,989	L_5m	0,985	L_5m	0,973	L_5m	0,923	L_5m	0,919	L_5m	0,928
L_5mv	0,981	L_5mv	0,987	L_5mv	0,960	L_5mv	0,920	L_5mv	0,901	L_5mv	0,923
L_23c	0,980	L_23c	0,982	L_23c	0,963	L_23c	0,924	L_23c	0,888	L_23c	0,899
L_5L	0,989	L_5L	0,988	L_5L	0,960	L_5L	0,906	L_5L	0,943	L_5L	0,928
L_24dd	0,987	L_24dd	0,987	L_24dd	0,971	L_24dd	0,940	L_24dd	0,926	L_24dd	0,928
L_24dv	0,978	L_24dv	0,985	L_24dv	0,969	L_24dv	0,911	L_24dv	0,907	L_24dv	0,913
L_7AL	0,987	L_7AL	0,989	L_7AL	0,968	L_7AL	0,896	L_7AL	0,964	L_7AL	0,925
L_SCEF	0,989	L_SCEF	0,985	L_SCEF	0,966	L_SCEF	0,934	L_SCEF	0,899	L_SCEF	0,935
L_6ma	0,988	L_6ma	0,983	L_6ma	0,958	L_6ma	0,926	L_6ma	0,905	L_6ma	0,933
L_7Am	0,989	L_7Am	0,988	L_7Am	0,968	L_7Am	0,908	L_7Am	0,910	L_7Am	0,912
L_7PL	0,989	L_7PL	0,989	L_7PL	0,968	L_7PL	0,901	L_7PL	0,949	L_7PL	0,926
L_7PC	0,988	L_7PC	0,990	L_7PC	0,965	L_7PC	0,915	L_7PC	0,924	L_7PC	0,935
L_LIPv	0,986	L_LIPv	0,989	L_LIPv	0,966	L_LIPv	0,905	L_LIPv	0,910	L_LIPv	0,934
L_VIP	0,987	L_VIP	0,988	L_VIP	0,966	L_VIP	0,904	L_VIP	0,959	L_VIP	0,934
L_MIP	0,986	L_MIP	0,987	L_MIP	0,971	L_MIP	0,907	L_MIP	0,920	L_MIP	0,929
L_1	0,992	L_1	0,988	L_1	0,972	L_1	0,935	L_1	0,916	L_1	0,950
L_2	0,991	L_2	0,989	L_2	0,970	L_2	0,932	L_2	0,921	L_2	0,941
L_3a	0,992	L_3a	0,988	L_3a	0,966	L_3a	0,927	L_3a	0,923	L_3a	0,939
L_6d	0,990	L_6d	0,986	L_6d	0,966	L_6d	0,929	L_6d	0,940	L_6d	0,931
L_6mp	0,989	L_6mp	0,985	L_6mp	0,965	L_6mp	0,918	L_6mp	0,924	L_6mp	0,933

L_6v	0,988	L_6v	0,987	L_6v	0,968	L_6v	0,934	L_6v	0,877	L_6v	0,932
L_p24pr	0,975	L_p24pr	0,983	L_p24pr	0,966	L_p24pr	0,919	L_p24pr	0,909	L_p24pr	0,921
L_33pr	0,968	L_33pr	0,982	L_33pr	0,963	L_33pr	0,934	L_33pr	0,931	L_33pr	0,921
L_a24pr	0,974	L_a24pr	0,985	L_a24pr	0,965	L_a24pr	0,926	L_a24pr	0,903	L_a24pr	0,924
L_p32pr	0,974	L_p32pr	0,987	L_p32pr	0,965	L_p32pr	0,941	L_p32pr	0,916	L_p32pr	0,922
L_a24	0,982	L_a24	0,984	L_a24	0,965	L_a24	0,901	L_a24	0,919	L_a24	0,938
L_d32	0,986	L_d32	0,985	L_d32	0,960	L_d32	0,914	L_d32	0,932	L_d32	0,933
L_8BM	0,989	L_8BM	0,984	L_8BM	0,961	L_8BM	0,917	L_8BM	0,928	L_8BM	0,942
L_p32	0,982	L_p32	0,984	L_p32	0,967	L_p32	0,898	L_p32	0,923	L_p32	0,929
L_10r	0,985	L_10r	0,982	L_10r	0,960	L_10r	0,910	L_10r	0,922	L_10r	0,940
L_47m	0,980	L_47m	0,974	L_47m	0,942	L_47m	0,903	L_47m	0,900	L_47m	0,906
L_8Av	0,989	L_8Av	0,984	L_8Av	0,959	L_8Av	0,906	L_8Av	0,916	L_8Av	0,936
L_8Ad	0,986	L_8Ad	0,983	L_8Ad	0,951	L_8Ad	0,880	L_8Ad	0,899	L_8Ad	0,925
L_9m	0,988	L_9m	0,985	L_9m	0,960	L_9m	0,917	L_9m	0,924	L_9m	0,942
L_8BL	0,988	L_8BL	0,985	L_8BL	0,946	L_8BL	0,905	L_8BL	0,915	L_8BL	0,951
L_9p	0,987	L_9p	0,980	L_9p	0,958	L_9p	0,888	L_9p	0,925	L_9p	0,935
L_10d	0,988	L_10d	0,984	L_10d	0,967	L_10d	0,915	L_10d	0,923	L_10d	0,934
L_8C	0,989	L_8C	0,984	L_8C	0,963	L_8C	0,903	L_8C	0,938	L_8C	0,924
L_44	0,988	L_44	0,983	L_44	0,964	L_44	0,928	L_44	0,901	L_44	0,926
L_45	0,987	L_45	0,983	L_45	0,959	L_45	0,917	L_45	0,924	L_45	0,908
L_47l	0,987	L_47l	0,983	L_47l	0,971	L_47l	0,911	L_47l	0,909	L_47l	0,918
L_a47r	0,983	L_a47r	0,982	L_a47r	0,955	L_a47r	0,908	L_a47r	0,931	L_a47r	0,919
L_6r	0,986	L_6r	0,986	L_6r	0,967	L_6r	0,940	L_6r	0,897	L_6r	0,927
L_IFJa	0,980	L_IFJa	0,984	L_IFJa	0,971	L_IFJa	0,922	L_IFJa	0,927	L_IFJa	0,930
L_IFJp	0,984	L_IFJp	0,981	L_IFJp	0,968	L_IFJp	0,931	L_IFJp	0,915	L_IFJp	0,930
L_IFSp	0,986	L_IFSp	0,985	L_IFSp	0,965	L_IFSp	0,918	L_IFSp	0,921	L_IFSp	0,928
L_IFSa	0,987	L_IFSa	0,983	L_IFSa	0,962	L_IFSa	0,906	L_IFSa	0,908	L_IFSa	0,924
L_p9.46v	0,987	L_p9.46v	0,985	L_p9.46v	0,959	L_p9.46v	0,905	L_p9.46v	0,901	L_p9.46v	0,933
L_46	0,988	L_46	0,982	L_46	0,954	L_46	0,915	L_46	0,894	L_46	0,933
L_a9.46v	0,988	L_a9.46v	0,981	L_a9.46v	0,961	L_a9.46v	0,903	L_a9.46v	0,927	L_a9.46v	0,929

L_9.46d	0,987	L_9.46d	0,982	L_9.46d	0,965	L_9.46d	0,914	L_9.46d	0,925	L_9.46d	0,920
L_9a	0,987	L_9a	0,985	L_9a	0,956	L_9a	0,925	L_9a	0,917	L_9a	0,929
L_10v	0,987	L_10v	0,980	L_10v	0,950	L_10v	0,921	L_10v	0,912	L_10v	0,930
L_a10p	0,985	L_a10p	0,981	L_a10p	0,961	L_a10p	0,913	L_a10p	0,931	L_a10p	0,915
L_10pp	0,990	L_10pp	0,979	L_10pp	0,962	L_10pp	0,900	L_10pp	0,933	L_10pp	0,912
L_11l	0,985	L_11l	0,976	L_11l	0,947	L_11l	0,896	L_11l	0,901	L_11l	0,918
L_13l	0,983	L_13l	0,973	L_13l	0,958	L_13l	0,899	L_13l	0,917	L_13l	0,939
L_OFC	0,984	L_OFC	0,977	L_OFC	0,942	L_OFC	0,877	L_OFC	0,919	L_OFC	0,943
L_47s	0,981	L_47s	0,979	L_47s	0,957	L_47s	0,900	L_47s	0,925	L_47s	0,921
L_LIPd	0,981	L_LIPd	0,987	L_LIPd	0,971	L_LIPd	0,914	L_LIPd	0,922	L_LIPd	0,926
L_6a	0,986	L_6a	0,984	L_6a	0,961	L_6a	0,917	L_6a	0,904	L_6a	0,928
L_i6.8	0,987	L_i6.8	0,985	L_i6.8	0,958	L_i6.8	0,898	L_i6.8	0,920	L_i6.8	0,935
L_s6.8	0,988	L_s6.8	0,983	L_s6.8	0,938	L_s6.8	0,913	L_s6.8	0,916	L_s6.8	0,930
L_43	0,985	L_43	0,987	L_43	0,965	L_43	0,931	L_43	0,870	L_43	0,944
L_OP4	0,990	L_OP4	0,987	L_OP4	0,966	L_OP4	0,939	L_OP4	0,867	L_OP4	0,940
L_OP1	0,987	L_OP1	0,988	L_OP1	0,970	L_OP1	0,932	L_OP1	0,889	L_OP1	0,927
L_OP2.3	0,980	L_OP2.3	0,986	L_OP2.3	0,970	L_OP2.3	0,931	L_OP2.3	0,878	L_OP2.3	0,933
L_52	0,977	L_52	0,977	L_52	0,961	L_52	0,936	L_52	0,897	L_52	0,914
L_RI	0,983	L_RI	0,982	L_RI	0,963	L_RI	0,940	L_RI	0,891	L_RI	0,924
L_PFcm	0,988	L_PFcm	0,985	L_PFcm	0,967	L_PFcm	0,930	L_PFcm	0,875	L_PFcm	0,929
L_PoI2	0,984	L_PoI2	0,981	L_PoI2	0,957	L_PoI2	0,926	L_PoI2	0,897	L_PoI2	0,928
L_TA2	0,986	L_TA2	0,976	L_TA2	0,959	L_TA2	0,922	L_TA2	0,900	L_TA2	0,930
L_FOP4	0,978	L_FOP4	0,987	L_FOP4	0,970	L_FOP4	0,933	L_FOP4	0,898	L_FOP4	0,929
L_MI	0,977	L_MI	0,985	L_MI	0,962	L_MI	0,934	L_MI	0,912	L_MI	0,930
L_Pir	0,959	L_Pir	0,962	L_Pir	0,948	L_Pir	0,899	L_Pir	0,917	L_Pir	0,905
L_AVI	0,970	L_AVI	0,986	L_AVI	0,956	L_AVI	0,923	L_AVI	0,918	L_AVI	0,904
L_AAIC	0,972	L_AAIC	0,977	L_AAIC	0,963	L_AAIC	0,904	L_AAIC	0,932	L_AAIC	0,913
L_FOP1	0,983	L_FOP1	0,987	L_FOP1	0,971	L_FOP1	0,929	L_FOP1	0,878	L_FOP1	0,934
L_FOP3	0,959	L_FOP3	0,985	L_FOP3	0,956	L_FOP3	0,926	L_FOP3	0,876	L_FOP3	0,904
L_FOP2	0,966	L_FOP2	0,987	L_FOP2	0,965	L_FOP2	0,928	L_FOP2	0,873	L_FOP2	0,929

L_PFt	0,988	L_PFt	0,987	L_PFt	0,967	L_PFt	0,921	L_PFt	0,890	L_PFt	0,922
L_AIP	0,983	L_AIP	0,990	L_AIP	0,970	L_AIP	0,899	L_AIP	0,920	L_AIP	0,930
L_EC	0,980	L_EC	0,961	L_EC	0,938	L_EC	0,852	L_EC	0,873	L_EC	0,885
L_PreS	0,974	L_PreS	0,971	L_PreS	0,939	L_PreS	0,890	L_PreS	0,877	L_PreS	0,884
L_H	0,904	L_H	0,998	L_H	0,989	L_H	0,994	L_H	0,997	L_H	0,998
L_ProS	0,986	L_ProS	0,978	L_ProS	0,956	L_ProS	0,901	L_ProS	0,868	L_ProS	0,909
L_PeEc	0,984	L_PeEc	0,973	L_PeEc	0,949	L_PeEc	0,833	L_PeEc	0,887	L_PeEc	0,912
L_STGa	0,981	L_STGa	0,977	L_STGa	0,966	L_STGa	0,911	L_STGa	0,914	L_STGa	0,922
L_PBelt	0,991	L_PBelt	0,988	L_PBelt	0,972	L_PBelt	0,933	L_PBelt	0,905	L_PBelt	0,938
L_A5	0,990	L_A5	0,987	L_A5	0,976	L_A5	0,938	L_A5	0,887	L_A5	0,942
L_PHA1	0,974	L_PHA1	0,979	L_PHA1	0,951	L_PHA1	0,908	L_PHA1	0,902	L_PHA1	0,895
L_PHA3	0,974	L_PHA3	0,980	L_PHA3	0,952	L_PHA3	0,917	L_PHA3	0,898	L_PHA3	0,920
L_STSda	0,984	L_STSda	0,984	L_STSda	0,971	L_STSda	0,926	L_STSda	0,907	L_STSda	0,931
L_STSdp	0,986	L_STSdp	0,988	L_STSdp	0,974	L_STSdp	0,919	L_STSdp	0,928	L_STSdp	0,925
L_STSvp	0,984	L_STSvp	0,984	L_STSvp	0,967	L_STSvp	0,920	L_STSvp	0,922	L_STSvp	0,927
L_TGd	0,986	L_TGd	0,978	L_TGd	0,958	L_TGd	0,879	L_TGd	0,903	L_TGd	0,915
L_TE1a	0,985	L_TE1a	0,984	L_TE1a	0,960	L_TE1a	0,910	L_TE1a	0,892	L_TE1a	0,928
L_TE1p	0,989	L_TE1p	0,985	L_TE1p	0,971	L_TE1p	0,928	L_TE1p	0,894	L_TE1p	0,928
L_TE2a	0,984	L_TE2a	0,974	L_TE2a	0,956	L_TE2a	0,899	L_TE2a	0,886	L_TE2a	0,923
L_TF	0,984	L_TF	0,977	L_TF	0,959	L_TF	0,884	L_TF	0,895	L_TF	0,909
L_TE2p	0,986	L_TE2p	0,983	L_TE2p	0,962	L_TE2p	0,921	L_TE2p	0,920	L_TE2p	0,919
L_PHT	0,988	L_PHT	0,986	L_PHT	0,966	L_PHT	0,922	L_PHT	0,907	L_PHT	0,925
L_PH	0,987	L_PH	0,987	L_PH	0,957	L_PH	0,919	L_PH	0,915	L_PH	0,931
L_TPOJ1	0,988	L_TPOJ1	0,985	L_TPOJ1	0,970	L_TPOJ1	0,939	L_TPOJ1	0,916	L_TPOJ1	0,923
L_TPOJ2	0,989	L_TPOJ2	0,985	L_TPOJ2	0,967	L_TPOJ2	0,912	L_TPOJ2	0,892	L_TPOJ2	0,924
L_TPOJ3	0,986	L_TPOJ3	0,983	L_TPOJ3	0,974	L_TPOJ3	0,907	L_TPOJ3	0,881	L_TPOJ3	0,921
L_DVT	0,988	L_DVT	0,987	L_DVT	0,961	L_DVT	0,915	L_DVT	0,899	L_DVT	0,936
L_PGp	0,986	L_PGp	0,985	L_PGp	0,961	L_PGp	0,910	L_PGp	0,894	L_PGp	0,912
L_IP2	0,982	L_IP2	0,986	L_IP2	0,971	L_IP2	0,923	L_IP2	0,916	L_IP2	0,927
L_IP1	0,986	L_IP1	0,986	L_IP1	0,964	L_IP1	0,889	L_IP1	0,924	L_IP1	0,934

L_IP0	0,984	L_IP0	0,987	L_IP0	0,965	L_IP0	0,909	L_IP0	0,926	L_IP0	0,923
L_PFop	0,989	L_PFop	0,987	L_PFop	0,959	L_PFop	0,931	L_PFop	0,871	L_PFop	0,934
L_PF	0,989	L_PF	0,982	L_PF	0,963	L_PF	0,932	L_PF	0,904	L_PF	0,935
L_PFm	0,989	L_PFm	0,984	L_PFm	0,964	L_PFm	0,907	L_PFm	0,931	L_PFm	0,937
L_PGi	0,987	L_PGi	0,982	L_PGi	0,963	L_PGi	0,910	L_PGi	0,915	L_PGi	0,936
L_PGs	0,988	L_PGs	0,984	L_PGs	0,966	L_PGs	0,901	L_PGs	0,912	L_PGs	0,945
L_V6A	0,990	L_V6A	0,987	L_V6A	0,968	L_V6A	0,899	L_V6A	0,918	L_V6A	0,932
L_VMV1	0,978	L_VMV1	0,983	L_VMV1	0,960	L_VMV1	0,910	L_VMV1	0,870	L_VMV1	0,922
L_VMV3	0,981	L_VMV3	0,984	L_VMV3	0,960	L_VMV3	0,915	L_VMV3	0,891	L_VMV3	0,913
L_PHA2	0,962	L_PHA2	0,979	L_PHA2	0,948	L_PHA2	0,881	L_PHA2	0,869	L_PHA2	0,913
L_V4t	0,989	L_V4t	0,988	L_V4t	0,972	L_V4t	0,923	L_V4t	0,926	L_V4t	0,924
L_FST	0,988	L_FST	0,989	L_FST	0,961	L_FST	0,915	L_FST	0,903	L_FST	0,926
L_V3CD	0,988	L_V3CD	0,987	L_V3CD	0,970	L_V3CD	0,900	L_V3CD	0,906	L_V3CD	0,929
L_LO3	0,987	L_LO3	0,987	L_LO3	0,964	L_LO3	0,901	L_LO3	0,914	L_LO3	0,931
L_VMV2	0,977	L_VMV2	0,984	L_VMV2	0,951	L_VMV2	0,916	L_VMV2	0,889	L_VMV2	0,922
L_31pd	0,976	L_31pd	0,982	L_31pd	0,961	L_31pd	0,900	L_31pd	0,900	L_31pd	0,930
L_31a	0,979	L_31a	0,983	L_31a	0,962	L_31a	0,897	L_31a	0,910	L_31a	0,907
L_VVC	0,985	L_VVC	0,983	L_VVC	0,960	L_VVC	0,917	L_VVC	0,900	L_VVC	0,920
L_25	0,971	L_25	0,977	L_25	0,955	L_25	0,885	L_25	0,902	L_25	0,916
L_s32	0,977	L_s32	0,980	L_s32	0,955	L_s32	0,910	L_s32	0,918	L_s32	0,955
L_pOFC	0,970	L_pOFC	0,972	L_pOFC	0,957	L_pOFC	0,885	L_pOFC	0,904	L_pOFC	0,913
L_PoI1	0,976	L_PoI1	0,977	L_PoI1	0,958	L_PoI1	0,917	L_PoI1	0,906	L_PoI1	0,917
L_Ig	0,976	L_Ig	0,985	L_Ig	0,967	L_Ig	0,924	L_Ig	0,885	L_Ig	0,922
L_FOP5	0,968	L_FOP5	0,985	L_FOP5	0,959	L_FOP5	0,930	L_FOP5	0,908	L_FOP5	0,902
L_p10p	0,989	L_p10p	0,980	L_p10p	0,963	L_p10p	0,896	L_p10p	0,944	L_p10p	0,915
L_p47r	0,986	L_p47r	0,981	L_p47r	0,954	L_p47r	0,887	L_p47r	0,927	L_p47r	0,911
L_TGv	0,986	L_TGv	0,976	L_TGv	0,963	L_TGv	0,876	L_TGv	0,898	L_TGv	0,897
L_MBelt	0,986	L_MBelt	0,984	L_MBelt	0,971	L_MBelt	0,920	L_MBelt	0,905	L_MBelt	0,937
L_LBelt	0,990	L_LBelt	0,986	L_LBelt	0,970	L_LBelt	0,940	L_LBelt	0,915	L_LBelt	0,935
L_A4	0,990	L_A4	0,986	L_A4	0,970	L_A4	0,938	L_A4	0,895	L_A4	0,946

L_STSva	0,984	L_STSva	0,981	L_STSva	0,964	L_STSva	0,899	L_STSva	0,916	L_STSva	0,924
L_TE1m	0,987	L_TE1m	0,983	L_TE1m	0,965	L_TE1m	0,921	L_TE1m	0,896	L_TE1m	0,925
L_PI	0,979	L_PI	0,960	L_PI	0,948	L_PI	0,856	L_PI	0,893	L_PI	0,898
L_a32pr	0,986	L_a32pr	0,985	L_a32pr	0,966	L_a32pr	0,916	L_a32pr	0,939	L_a32pr	0,935
L_p24	0,976	L_p24	0,982	L_p24	0,964	L_p24	0,916	L_p24	0,924	L_p24	0,930
R_V1	0,990	R_V1	0,985	R_V1	0,960	R_V1	0,926	R_V1	0,909	R_V1	0,920
R_MST	0,989	R_MST	0,986	R_MST	0,971	R_MST	0,915	R_MST	0,876	R_MST	0,922
R_V6	0,989	R_V6	0,981	R_V6	0,959	R_V6	0,919	R_V6	0,901	R_V6	0,931
R_V2	0,990	R_V2	0,985	R_V2	0,968	R_V2	0,910	R_V2	0,904	R_V2	0,938
R_V3	0,991	R_V3	0,987	R_V3	0,974	R_V3	0,912	R_V3	0,909	R_V3	0,946
R_V4	0,991	R_V4	0,989	R_V4	0,972	R_V4	0,922	R_V4	0,886	R_V4	0,936
R_V8	0,990	R_V8	0,986	R_V8	0,966	R_V8	0,919	R_V8	0,899	R_V8	0,918
R_4	0,993	R_4	0,989	R_4	0,973	R_4	0,937	R_4	0,922	R_4	0,944
R_3b	0,992	R_3b	0,989	R_3b	0,972	R_3b	0,937	R_3b	0,923	R_3b	0,936
R_FEF	0,986	R_FEF	0,979	R_FEF	0,966	R_FEF	0,913	R_FEF	0,911	R_FEF	0,923
R_PEF	0,985	R_PEF	0,985	R_PEF	0,961	R_PEF	0,918	R_PEF	0,913	R_PEF	0,930
R_55b	0,989	R_55b	0,985	R_55b	0,967	R_55b	0,914	R_55b	0,912	R_55b	0,929
R_V3A	0,991	R_V3A	0,985	R_V3A	0,972	R_V3A	0,917	R_V3A	0,901	R_V3A	0,943
R_RSC	0,979	R_RSC	0,983	R_RSC	0,959	R_RSC	0,895	R_RSC	0,888	R_RSC	0,895
R_POS2	0,988	R_POS2	0,979	R_POS2	0,965	R_POS2	0,896	R_POS2	0,921	R_POS2	0,931
R_V7	0,989	R_V7	0,988	R_V7	0,970	R_V7	0,916	R_V7	0,892	R_V7	0,938
R_IPS1	0,987	R_IPS1	0,988	R_IPS1	0,971	R_IPS1	0,917	R_IPS1	0,925	R_IPS1	0,934
R_FFC	0,990	R_FFC	0,982	R_FFC	0,970	R_FFC	0,933	R_FFC	0,893	R_FFC	0,919
R_V3B	0,984	R_V3B	0,988	R_V3B	0,967	R_V3B	0,904	R_V3B	0,913	R_V3B	0,924
R_LO1	0,988	R_LO1	0,987	R_LO1	0,965	R_LO1	0,903	R_LO1	0,894	R_LO1	0,933
R_LO2	0,988	R_LO2	0,989	R_LO2	0,970	R_LO2	0,913	R_LO2	0,919	R_LO2	0,930
R_PIT	0,989	R_PIT	0,985	R_PIT	0,963	R_PIT	0,910	R_PIT	0,910	R_PIT	0,922
R_MT	0,990	R_MT	0,988	R_MT	0,967	R_MT	0,913	R_MT	0,904	R_MT	0,933
R_A1	0,984	R_A1	0,984	R_A1	0,970	R_A1	0,903	R_A1	0,919	R_A1	0,952
R_PSL	0,988	R_PSL	0,985	R_PSL	0,949	R_PSL	0,916	R_PSL	0,913	R_PSL	0,933

R_SFL	0,987	R_SFL	0,981	R_SFL	0,960	R_SFL	0,921	R_SFL	0,910	R_SFL	0,941
R_PCV	0,986	R_PCV	0,983	R_PCV	0,966	R_PCV	0,895	R_PCV	0,907	R_PCV	0,918
R_STV	0,988	R_STV	0,986	R_STV	0,970	R_STV	0,929	R_STV	0,912	R_STV	0,942
R_7Pm	0,986	R_7Pm	0,987	R_7Pm	0,966	R_7Pm	0,909	R_7Pm	0,936	R_7Pm	0,928
R_7m	0,982	R_7m	0,982	R_7m	0,963	R_7m	0,895	R_7m	0,892	R_7m	0,934
R_POS1	0,985	R_POS1	0,980	R_POS1	0,951	R_POS1	0,903	R_POS1	0,894	R_POS1	0,917
R_23d	0,977	R_23d	0,980	R_23d	0,964	R_23d	0,892	R_23d	0,911	R_23d	0,914
R_v23ab	0,977	R_v23ab	0,983	R_v23ab	0,952	R_v23ab	0,907	R_v23ab	0,903	R_v23ab	0,919
R_d23ab	0,975	R_d23ab	0,984	R_d23ab	0,953	R_d23ab	0,890	R_d23ab	0,886	R_d23ab	0,923
R_31pv	0,978	R_31pv	0,983	R_31pv	0,957	R_31pv	0,890	R_31pv	0,900	R_31pv	0,914
R_5m	0,989	R_5m	0,987	R_5m	0,972	R_5m	0,924	R_5m	0,923	R_5m	0,935
R_5mv	0,984	R_5mv	0,986	R_5mv	0,963	R_5mv	0,914	R_5mv	0,887	R_5mv	0,908
R_23c	0,983	R_23c	0,981	R_23c	0,957	R_23c	0,911	R_23c	0,879	R_23c	0,910
R_5L	0,989	R_5L	0,988	R_5L	0,969	R_5L	0,900	R_5L	0,961	R_5L	0,933
R_24dd	0,989	R_24dd	0,983	R_24dd	0,971	R_24dd	0,938	R_24dd	0,922	R_24dd	0,934
R_24dv	0,982	R_24dv	0,987	R_24dv	0,966	R_24dv	0,931	R_24dv	0,912	R_24dv	0,917
R_7AL	0,987	R_7AL	0,987	R_7AL	0,960	R_7AL	0,898	R_7AL	0,955	R_7AL	0,913
R_SCEF	0,988	R_SCEF	0,983	R_SCEF	0,969	R_SCEF	0,937	R_SCEF	0,890	R_SCEF	0,940
R_6ma	0,987	R_6ma	0,984	R_6ma	0,960	R_6ma	0,917	R_6ma	0,908	R_6ma	0,927
R_7Am	0,988	R_7Am	0,988	R_7Am	0,961	R_7Am	0,909	R_7Am	0,911	R_7Am	0,921
R_7PL	0,986	R_7PL	0,989	R_7PL	0,964	R_7PL	0,909	R_7PL	0,937	R_7PL	0,933
R_7PC	0,990	R_7PC	0,987	R_7PC	0,969	R_7PC	0,931	R_7PC	0,932	R_7PC	0,930
R_LIPv	0,987	R_LIPv	0,989	R_LIPv	0,967	R_LIPv	0,909	R_LIPv	0,914	R_LIPv	0,927
R_VIP	0,988	R_VIP	0,988	R_VIP	0,968	R_VIP	0,898	R_VIP	0,946	R_VIP	0,935
R_MIP	0,986	R_MIP	0,988	R_MIP	0,971	R_MIP	0,911	R_MIP	0,912	R_MIP	0,926
R_1	0,992	R_1	0,988	R_1	0,971	R_1	0,933	R_1	0,913	R_1	0,944
R_2	0,992	R_2	0,989	R_2	0,973	R_2	0,938	R_2	0,909	R_2	0,946
R_3a	0,992	R_3a	0,989	R_3a	0,974	R_3a	0,931	R_3a	0,927	R_3a	0,946
R_6d	0,990	R_6d	0,988	R_6d	0,969	R_6d	0,923	R_6d	0,903	R_6d	0,931
R_6mp	0,990	R_6mp	0,987	R_6mp	0,968	R_6mp	0,912	R_6mp	0,925	R_6mp	0,929

R_6v	0,990	R_6v	0,984	R_6v	0,967	R_6v	0,926	R_6v	0,904	R_6v	0,913
R_p24pr	0,979	R_p24pr	0,984	R_p24pr	0,961	R_p24pr	0,929	R_p24pr	0,901	R_p24pr	0,914
R_33pr	0,971	R_33pr	0,981	R_33pr	0,960	R_33pr	0,910	R_33pr	0,911	R_33pr	0,911
R_a24pr	0,975	R_a24pr	0,988	R_a24pr	0,962	R_a24pr	0,929	R_a24pr	0,911	R_a24pr	0,922
R_p32pr	0,982	R_p32pr	0,986	R_p32pr	0,962	R_p32pr	0,940	R_p32pr	0,911	R_p32pr	0,926
R_a24	0,977	R_a24	0,984	R_a24	0,960	R_a24	0,900	R_a24	0,922	R_a24	0,933
R_d32	0,984	R_d32	0,988	R_d32	0,960	R_d32	0,911	R_d32	0,931	R_d32	0,936
R_8BM	0,986	R_8BM	0,984	R_8BM	0,964	R_8BM	0,925	R_8BM	0,933	R_8BM	0,945
R_p32	0,980	R_p32	0,983	R_p32	0,957	R_p32	0,908	R_p32	0,928	R_p32	0,935
R_10r	0,981	R_10r	0,983	R_10r	0,953	R_10r	0,924	R_10r	0,910	R_10r	0,941
R_47m	0,983	R_47m	0,970	R_47m	0,954	R_47m	0,894	R_47m	0,905	R_47m	0,922
R_8Av	0,986	R_8Av	0,985	R_8Av	0,961	R_8Av	0,922	R_8Av	0,944	R_8Av	0,944
R_8Ad	0,984	R_8Ad	0,985	R_8Ad	0,951	R_8Ad	0,893	R_8Ad	0,899	R_8Ad	0,936
R_9m	0,987	R_9m	0,984	R_9m	0,956	R_9m	0,910	R_9m	0,919	R_9m	0,932
R_8BL	0,986	R_8BL	0,983	R_8BL	0,956	R_8BL	0,900	R_8BL	0,931	R_8BL	0,949
R_9p	0,986	R_9p	0,978	R_9p	0,953	R_9p	0,900	R_9p	0,921	R_9p	0,937
R_10d	0,989	R_10d	0,982	R_10d	0,967	R_10d	0,918	R_10d	0,911	R_10d	0,933
R_8C	0,988	R_8C	0,984	R_8C	0,967	R_8C	0,905	R_8C	0,941	R_8C	0,936
R_44	0,987	R_44	0,985	R_44	0,967	R_44	0,926	R_44	0,913	R_44	0,928
R_45	0,987	R_45	0,986	R_45	0,970	R_45	0,927	R_45	0,913	R_45	0,928
R_47l	0,987	R_47l	0,982	R_47l	0,970	R_47l	0,928	R_47l	0,921	R_47l	0,930
R_a47r	0,986	R_a47r	0,978	R_a47r	0,957	R_a47r	0,908	R_a47r	0,917	R_a47r	0,934
R_6r	0,988	R_6r	0,986	R_6r	0,964	R_6r	0,934	R_6r	0,912	R_6r	0,933
R_IFJa	0,978	R_IFJa	0,983	R_IFJa	0,958	R_IFJa	0,915	R_IFJa	0,919	R_IFJa	0,934
R_IFJp	0,979	R_IFJp	0,983	R_IFJp	0,962	R_IFJp	0,910	R_IFJp	0,920	R_IFJp	0,940
R_IFSp	0,987	R_IFSp	0,985	R_IFSp	0,965	R_IFSp	0,907	R_IFSp	0,925	R_IFSp	0,933
R_IFSa	0,985	R_IFSa	0,975	R_IFSa	0,964	R_IFSa	0,903	R_IFSa	0,914	R_IFSa	0,932
R_p9.46v	0,987	R_p9.46v	0,982	R_p9.46v	0,962	R_p9.46v	0,911	R_p9.46v	0,933	R_p9.46v	0,936
R_46	0,988	R_46	0,982	R_46	0,951	R_46	0,911	R_46	0,925	R_46	0,938
R_a9.46v	0,989	R_a9.46v	0,972	R_a9.46v	0,962	R_a9.46v	0,907	R_a9.46v	0,923	R_a9.46v	0,936

R_9.46d	0,988	R_9.46d	0,984	R_9.46d	0,951	R_9.46d	0,914	R_9.46d	0,927	R_9.46d	0,931
R_9a	0,987	R_9a	0,981	R_9a	0,950	R_9a	0,909	R_9a	0,920	R_9a	0,931
R_10v	0,988	R_10v	0,985	R_10v	0,961	R_10v	0,914	R_10v	0,908	R_10v	0,933
R_a10p	0,986	R_a10p	0,983	R_a10p	0,967	R_a10p	0,910	R_a10p	0,918	R_a10p	0,926
R_10pp	0,989	R_10pp	0,982	R_10pp	0,966	R_10pp	0,907	R_10pp	0,941	R_10pp	0,925
R_11l	0,986	R_11l	0,975	R_11l	0,955	R_11l	0,895	R_11l	0,897	R_11l	0,933
R_13l	0,985	R_13l	0,973	R_13l	0,955	R_13l	0,903	R_13l	0,912	R_13l	0,940
R_OFC	0,981	R_OFC	0,976	R_OFC	0,959	R_OFC	0,912	R_OFC	0,921	R_OFC	0,945
R_47s	0,985	R_47s	0,974	R_47s	0,956	R_47s	0,912	R_47s	0,925	R_47s	0,912
R_LIPd	0,985	R_LIPd	0,987	R_LIPd	0,969	R_LIPd	0,916	R_LIPd	0,902	R_LIPd	0,937
R_6a	0,984	R_6a	0,985	R_6a	0,955	R_6a	0,907	R_6a	0,897	R_6a	0,918
R_i6.8	0,985	R_i6.8	0,986	R_i6.8	0,964	R_i6.8	0,909	R_i6.8	0,934	R_i6.8	0,933
R_s6.8	0,986	R_s6.8	0,985	R_s6.8	0,955	R_s6.8	0,907	R_s6.8	0,926	R_s6.8	0,940
R_43	0,989	R_43	0,987	R_43	0,967	R_43	0,939	R_43	0,904	R_43	0,938
R_OP4	0,992	R_OP4	0,989	R_OP4	0,967	R_OP4	0,939	R_OP4	0,902	R_OP4	0,943
R_OP1	0,987	R_OP1	0,988	R_OP1	0,972	R_OP1	0,930	R_OP1	0,919	R_OP1	0,946
R_OP2.3	0,984	R_OP2.3	0,984	R_OP2.3	0,972	R_OP2.3	0,927	R_OP2.3	0,886	R_OP2.3	0,950
R_52	0,976	R_52	0,972	R_52	0,955	R_52	0,917	R_52	0,913	R_52	0,935
R_RI	0,987	R_RI	0,985	R_RI	0,971	R_RI	0,949	R_RI	0,925	R_RI	0,937
R_PFcm	0,990	R_PFcm	0,987	R_PFcm	0,963	R_PFcm	0,929	R_PFcm	0,916	R_PFcm	0,936
R_PoI2	0,987	R_PoI2	0,983	R_PoI2	0,959	R_PoI2	0,924	R_PoI2	0,886	R_PoI2	0,936
R_TA2	0,986	R_TA2	0,982	R_TA2	0,959	R_TA2	0,918	R_TA2	0,908	R_TA2	0,937
R_FOP4	0,979	R_FOP4	0,987	R_FOP4	0,963	R_FOP4	0,934	R_FOP4	0,905	R_FOP4	0,920
R_MI	0,979	R_MI	0,987	R_MI	0,961	R_MI	0,930	R_MI	0,894	R_MI	0,923
R_Pir	0,977	R_Pir	0,964	R_Pir	0,951	R_Pir	0,918	R_Pir	0,888	R_Pir	0,904
R_AVI	0,980	R_AVI	0,983	R_AVI	0,960	R_AVI	0,918	R_AVI	0,922	R_AVI	0,912
R_AAIC	0,981	R_AAIC	0,976	R_AAIC	0,959	R_AAIC	0,903	R_AAIC	0,921	R_AAIC	0,901
R_FOP1	0,985	R_FOP1	0,987	R_FOP1	0,966	R_FOP1	0,935	R_FOP1	0,876	R_FOP1	0,939
R_FOP3	0,972	R_FOP3	0,985	R_FOP3	0,962	R_FOP3	0,935	R_FOP3	0,871	R_FOP3	0,927
R_FOP2	0,981	R_FOP2	0,987	R_FOP2	0,960	R_FOP2	0,933	R_FOP2	0,885	R_FOP2	0,939

R_PFt	0,989	R_PFt	0,988	R_PFt	0,967	R_PFt	0,921	R_PFt	0,930	R_PFt	0,930
R_AIP	0,987	R_AIP	0,988	R_AIP	0,968	R_AIP	0,907	R_AIP	0,911	R_AIP	0,929
R_EC	0,968	R_EC	0,957	R_EC	0,916	R_EC	0,821	R_EC	0,842	R_EC	0,855
R_PreS	0,972	R_PreS	0,966	R_PreS	0,951	R_PreS	0,858	R_PreS	0,852	R_PreS	0,953
R_H	0,000	R_H	0,997	R_H	0,985	R_H	0,994	R_H	0,994	R_H	0,998
R_ProS	0,987	R_ProS	0,977	R_ProS	0,951	R_ProS	0,911	R_ProS	0,867	R_ProS	0,916
R_PeEc	0,971	R_PeEc	0,969	R_PeEc	0,951	R_PeEc	0,841	R_PeEc	0,869	R_PeEc	0,888
R_STGa	0,988	R_STGa	0,980	R_STGa	0,964	R_STGa	0,913	R_STGa	0,899	R_STGa	0,920
R_PBelt	0,992	R_PBelt	0,987	R_PBelt	0,973	R_PBelt	0,949	R_PBelt	0,908	R_PBelt	0,945
R_A5	0,992	R_A5	0,986	R_A5	0,975	R_A5	0,938	R_A5	0,896	R_A5	0,936
R_PHA1	0,976	R_PHA1	0,978	R_PHA1	0,953	R_PHA1	0,897	R_PHA1	0,880	R_PHA1	0,896
R_PHA3	0,960	R_PHA3	0,981	R_PHA3	0,950	R_PHA3	0,904	R_PHA3	0,902	R_PHA3	0,917
R_STSda	0,989	R_STSda	0,984	R_STSda	0,970	R_STSda	0,919	R_STSda	0,908	R_STSda	0,920
R_STSdp	0,990	R_STSdp	0,986	R_STSdp	0,972	R_STSdp	0,934	R_STSdp	0,912	R_STSdp	0,940
R_STSvp	0,986	R_STSvp	0,984	R_STSvp	0,963	R_STSvp	0,910	R_STSvp	0,900	R_STSvp	0,926
R_TGd	0,986	R_TGd	0,982	R_TGd	0,962	R_TGd	0,889	R_TGd	0,887	R_TGd	0,921
R_TE1a	0,987	R_TE1a	0,983	R_TE1a	0,957	R_TE1a	0,890	R_TE1a	0,895	R_TE1a	0,925
R_TE1p	0,989	R_TE1p	0,980	R_TE1p	0,964	R_TE1p	0,918	R_TE1p	0,914	R_TE1p	0,926
R_TE2a	0,983	R_TE2a	0,979	R_TE2a	0,959	R_TE2a	0,880	R_TE2a	0,896	R_TE2a	0,924
R_TF	0,976	R_TF	0,970	R_TF	0,953	R_TF	0,890	R_TF	0,912	R_TF	0,937
R_TE2p	0,977	R_TE2p	0,977	R_TE2p	0,958	R_TE2p	0,904	R_TE2p	0,908	R_TE2p	0,909
R_PHT	0,990	R_PHT	0,981	R_PHT	0,964	R_PHT	0,909	R_PHT	0,918	R_PHT	0,933
R_PH	0,990	R_PH	0,986	R_PH	0,966	R_PH	0,922	R_PH	0,907	R_PH	0,925
R_TPOJ1	0,991	R_TPOJ1	0,986	R_TPOJ1	0,977	R_TPOJ1	0,930	R_TPOJ1	0,900	R_TPOJ1	0,938
R_TPOJ2	0,990	R_TPOJ2	0,986	R_TPOJ2	0,968	R_TPOJ2	0,911	R_TPOJ2	0,917	R_TPOJ2	0,927
R_TPOJ3	0,984	R_TPOJ3	0,983	R_TPOJ3	0,966	R_TPOJ3	0,909	R_TPOJ3	0,888	R_TPOJ3	0,926
R_DVT	0,987	R_DVT	0,985	R_DVT	0,953	R_DVT	0,926	R_DVT	0,915	R_DVT	0,928
R_PGp	0,988	R_PGp	0,985	R_PGp	0,957	R_PGp	0,915	R_PGp	0,883	R_PGp	0,921
R_IP2	0,986	R_IP2	0,987	R_IP2	0,967	R_IP2	0,917	R_IP2	0,929	R_IP2	0,929
R_IP1	0,989	R_IP1	0,988	R_IP1	0,970	R_IP1	0,908	R_IP1	0,926	R_IP1	0,941

R_IP0	0,985	R_IP0	0,989	R_IP0	0,961	R_IP0	0,906	R_IP0	0,922	R_IP0	0,926
R_PFop	0,990	R_PFop	0,988	R_PFop	0,964	R_PFop	0,931	R_PFop	0,887	R_PFop	0,935
R_PF	0,988	R_PF	0,984	R_PF	0,966	R_PF	0,926	R_PF	0,901	R_PF	0,924
R_PFm	0,989	R_PFm	0,984	R_PFm	0,967	R_PFm	0,920	R_PFm	0,937	R_PFm	0,944
R_PGi	0,988	R_PGi	0,985	R_PGi	0,963	R_PGi	0,911	R_PGi	0,905	R_PGi	0,924
R_PGs	0,989	R_PGs	0,983	R_PGs	0,964	R_PGs	0,909	R_PGs	0,930	R_PGs	0,935
R_V6A	0,989	R_V6A	0,985	R_V6A	0,961	R_V6A	0,917	R_V6A	0,918	R_V6A	0,928
R_VMV1	0,984	R_VMV1	0,981	R_VMV1	0,959	R_VMV1	0,918	R_VMV1	0,870	R_VMV1	0,928
R_VMV3	0,981	R_VMV3	0,986	R_VMV3	0,963	R_VMV3	0,930	R_VMV3	0,874	R_VMV3	0,917
R_PHA2	0,963	R_PHA2	0,980	R_PHA2	0,946	R_PHA2	0,879	R_PHA2	0,902	R_PHA2	0,905
R_V4t	0,990	R_V4t	0,988	R_V4t	0,973	R_V4t	0,922	R_V4t	0,933	R_V4t	0,929
R_FST	0,989	R_FST	0,988	R_FST	0,967	R_FST	0,902	R_FST	0,906	R_FST	0,921
R_V3CD	0,989	R_V3CD	0,988	R_V3CD	0,967	R_V3CD	0,904	R_V3CD	0,902	R_V3CD	0,928
R_LO3	0,990	R_LO3	0,986	R_LO3	0,965	R_LO3	0,907	R_LO3	0,879	R_LO3	0,923
R_VMV2	0,980	R_VMV2	0,984	R_VMV2	0,957	R_VMV2	0,920	R_VMV2	0,901	R_VMV2	0,928
R_31pd	0,977	R_31pd	0,979	R_31pd	0,956	R_31pd	0,904	R_31pd	0,903	R_31pd	0,923
R_31a	0,979	R_31a	0,977	R_31a	0,958	R_31a	0,889	R_31a	0,907	R_31a	0,909
R_VVC	0,985	R_VVC	0,983	R_VVC	0,967	R_VVC	0,926	R_VVC	0,893	R_VVC	0,921
R_25	0,970	R_25	0,973	R_25	0,946	R_25	0,875	R_25	0,876	R_25	0,907
R_s32	0,974	R_s32	0,977	R_s32	0,957	R_s32	0,908	R_s32	0,913	R_s32	0,956
R_pOFC	0,986	R_pOFC	0,975	R_pOFC	0,962	R_pOFC	0,883	R_pOFC	0,900	R_pOFC	0,908
R_PoI1	0,980	R_PoI1	0,976	R_PoI1	0,958	R_PoI1	0,920	R_PoI1	0,895	R_PoI1	0,934
R_Ig	0,979	R_Ig	0,983	R_Ig	0,969	R_Ig	0,934	R_Ig	0,889	R_Ig	0,937
R_FOP5	0,979	R_FOP5	0,985	R_FOP5	0,966	R_FOP5	0,928	R_FOP5	0,904	R_FOP5	0,922
R_p10p	0,989	R_p10p	0,977	R_p10p	0,954	R_p10p	0,901	R_p10p	0,928	R_p10p	0,922
R_p47r	0,986	R_p47r	0,978	R_p47r	0,962	R_p47r	0,902	R_p47r	0,902	R_p47r	0,933
R_TGv	0,982	R_TGv	0,976	R_TGv	0,966	R_TGv	0,879	R_TGv	0,931	R_TGv	0,903
R_MBelt	0,985	R_MBelt	0,983	R_MBelt	0,968	R_MBelt	0,928	R_MBelt	0,913	R_MBelt	0,949
R_LBelt	0,989	R_LBelt	0,987	R_LBelt	0,974	R_LBelt	0,939	R_LBelt	0,916	R_LBelt	0,947
R_A4	0,991	R_A4	0,987	R_A4	0,971	R_A4	0,944	R_A4	0,925	R_A4	0,931

R_STSva	0,984	R_STSva	0,984	R_STSva	0,964	R_STSva	0,900	R_STSva	0,895	R_STSva	0,923
R_TE1m	0,989	R_TE1m	0,981	R_TE1m	0,963	R_TE1m	0,912	R_TE1m	0,920	R_TE1m	0,932
R_PI	0,971	R_PI	0,958	R_PI	0,939	R_PI	0,865	R_PI	0,866	R_PI	0,908
R_a32pr	0,974	R_a32pr	0,988	R_a32pr	0,967	R_a32pr	0,932	R_a32pr	0,926	R_a32pr	0,926
R_p24	0,978	R_p24	0,987	R_p24	0,968	R_p24	0,928	R_p24	0,929	R_p24	0,926
L_accumbens	0,969	L_accumbens	0,966	L_accumbens	0,937	L_accumbens	0,896	L_accumbens	0,907	L_accumbens	0,882
L_amygdala	0,972	L_amygdala	0,955	L_amygdala	0,939	L_amygdala	0,889	L_amygdala	0,892	L_amygdala	0,882
L_caudate	0,984	L_caudate	0,972	L_caudate	0,965	L_caudate	0,911	L_caudate	0,924	L_caudate	0,917
L_hippocamp	0,985	L_hippocamp	0,960	L_hippocamp	0,947	L_hippocamp	0,885	L_hippocamp	0,879	L_hippocamp	0,884
L_pallidum	0,975	L_pallidum	0,964	L_pallidum	0,931	L_pallidum	0,892	L_pallidum	0,887	L_pallidum	0,813
L_putamen	0,986	L_putamen	0,976	L_putamen	0,954	L_putamen	0,928	L_putamen	0,911	L_putamen	0,877
L_thalamus	0,986	L_thalamus	0,967	L_thalamus	0,959	L_thalamus	0,932	L_thalamus	0,919	L_thalamus	0,876
L_ventraldc	0,980	L_ventraldc	0,959	L_ventraldc	0,940	L_ventraldc	0,907	L_ventraldc	0,879	L_ventraldc	0,863
R_accumbens	0,972	R_accumbens	0,969	R_accumbens	0,920	R_accumbens	0,892	R_accumbens	0,917	R_accumbens	0,876
R_amygdala	0,977	R_amygdala	0,963	R_amygdala	0,948	R_amygdala	0,903	R_amygdala	0,871	R_amygdala	0,870
R_caudate	0,983	R_caudate	0,975	R_caudate	0,953	R_caudate	0,910	R_caudate	0,916	R_caudate	0,901
R_hippocamp	0,982	R_hippocamp	0,958	R_hippocamp	0,929	R_hippocamp	0,891	R_hippocamp	0,868	R_hippocamp	0,879
R_pallidum	0,981	R_pallidum	0,960	R_pallidum	0,935	R_pallidum	0,885	R_pallidum	0,862	R_pallidum	0,796
R_putamen	0,986	R_putamen	0,979	R_putamen	0,951	R_putamen	0,926	R_putamen	0,909	R_putamen	0,899
R_thalamus	0,987	R_thalamus	0,970	R_thalamus	0,956	R_thalamus	0,920	R_thalamus	0,919	R_thalamus	0,897
R_ventraldc	0,983	R_ventraldc	0,959	R_ventraldc	0,937	R_ventraldc	0,903	R_ventraldc	0,868	R_ventraldc	0,840

Supplementary Table S11 Split-half reliability of all HCPMMP and subcortical areas (local clustering). Left hemispheric areas are labeled "L", right hemispheric areas are labeled "R".

HCP day 1 session 1		NKI (TR = 645)		NKI (TR = 1400)		NKI (TR = 2500)		UMN		RUB	
Area	SB	Area	SB	Area	SB	Area	SB	Area	SB	Area	SB
L_V1	0,924	L_V1	0,930	L_V1	0,912	L_V1	0,876	L_V1	0,832	L_V1	0,871
L_MST	0,889	L_MST	0,925	L_MST	0,924	L_MST	0,769	L_MST	0,768	L_MST	0,788
L_V6	0,905	L_V6	0,907	L_V6	0,876	L_V6	0,785	L_V6	0,807	L_V6	0,757
L_V2	0,915	L_V2	0,924	L_V2	0,911	L_V2	0,850	L_V2	0,817	L_V2	0,851
L_V3	0,919	L_V3	0,935	L_V3	0,921	L_V3	0,855	L_V3	0,817	L_V3	0,847
L_V4	0,921	L_V4	0,947	L_V4	0,908	L_V4	0,839	L_V4	0,811	L_V4	0,868
L_V8	0,889	L_V8	0,908	L_V8	0,864	L_V8	0,814	L_V8	0,815	L_V8	0,850
L_4	0,899	L_4	0,936	L_4	0,914	L_4	0,812	L_4	0,847	L_4	0,852
L_3b	0,900	L_3b	0,917	L_3b	0,879	L_3b	0,839	L_3b	0,809	L_3b	0,859
L_FEF	0,849	L_FEF	0,865	L_FEF	0,775	L_FEF	0,722	L_FEF	0,621	L_FEF	0,674
L_PEF	0,855	L_PEF	0,860	L_PEF	0,818	L_PEF	0,745	L_PEF	0,735	L_PEF	0,748
L_55b	0,879	L_55b	0,891	L_55b	0,852	L_55b	0,738	L_55b	0,695	L_55b	0,677
L_V3A	0,928	L_V3A	0,932	L_V3A	0,913	L_V3A	0,847	L_V3A	0,842	L_V3A	0,859
L_RSC	0,850	L_RSC	0,878	L_RSC	0,859	L_RSC	0,770	L_RSC	0,684	L_RSC	0,711
L_POS2	0,867	L_POS2	0,911	L_POS2	0,857	L_POS2	0,683	L_POS2	0,807	L_POS2	0,772
L_V7	0,904	L_V7	0,924	L_V7	0,874	L_V7	0,758	L_V7	0,778	L_V7	0,835
L_IPS1	0,864	L_IPS1	0,910	L_IPS1	0,884	L_IPS1	0,809	L_IPS1	0,755	L_IPS1	0,798
L_FFC	0,882	L_FFC	0,903	L_FFC	0,869	L_FFC	0,767	L_FFC	0,781	L_FFC	0,830
L_V3B	0,868	L_V3B	0,917	L_V3B	0,835	L_V3B	0,782	L_V3B	0,773	L_V3B	0,811
L_LO1	0,919	L_LO1	0,937	L_LO1	0,909	L_LO1	0,796	L_LO1	0,814	L_LO1	0,860
L_LO2	0,934	L_LO2	0,938	L_LO2	0,906	L_LO2	0,812	L_LO2	0,832	L_LO2	0,863
L_PIT	0,881	L_PIT	0,947	L_PIT	0,898	L_PIT	0,758	L_PIT	0,765	L_PIT	0,833
L_MT	0,881	L_MT	0,933	L_MT	0,911	L_MT	0,826	L_MT	0,804	L_MT	0,809
L_A1	0,882	L_A1	0,936	L_A1	0,876	L_A1	0,784	L_A1	0,782	L_A1	0,778
L_PSL	0,854	L_PSL	0,877	L_PSL	0,833	L_PSL	0,709	L_PSL	0,677	L_PSL	0,736

L_SFL	0,892	L_SFL	0,864	L_SFL	0,836	L_SFL	0,751	L_SFL	0,770	L_SFL	0,777
L_PCV	0,862	L_PCV	0,915	L_PCV	0,845	L_PCV	0,716	L_PCV	0,757	L_PCV	0,764
L_STV	0,871	L_STV	0,894	L_STV	0,868	L_STV	0,754	L_STV	0,726	L_STV	0,786
L_7Pm	0,887	L_7Pm	0,906	L_7Pm	0,834	L_7Pm	0,752	L_7Pm	0,776	L_7Pm	0,803
L_7m	0,885	L_7m	0,912	L_7m	0,879	L_7m	0,816	L_7m	0,812	L_7m	0,824
L_POS1	0,865	L_POS1	0,880	L_POS1	0,862	L_POS1	0,773	L_POS1	0,749	L_POS1	0,729
L_23d	0,858	L_23d	0,878	L_23d	0,852	L_23d	0,730	L_23d	0,710	L_23d	0,737
L_v23ab	0,866	L_v23ab	0,889	L_v23ab	0,842	L_v23ab	0,826	L_v23ab	0,772	L_v23ab	0,761
L_d23ab	0,876	L_d23ab	0,904	L_d23ab	0,861	L_d23ab	0,786	L_d23ab	0,759	L_d23ab	0,734
L_31pv	0,855	L_31pv	0,881	L_31pv	0,857	L_31pv	0,822	L_31pv	0,743	L_31pv	0,737
L_5m	0,900	L_5m	0,929	L_5m	0,901	L_5m	0,803	L_5m	0,789	L_5m	0,851
L_5mv	0,864	L_5mv	0,884	L_5mv	0,855	L_5mv	0,680	L_5mv	0,773	L_5mv	0,753
L_23c	0,857	L_23c	0,871	L_23c	0,799	L_23c	0,716	L_23c	0,685	L_23c	0,692
L_5L	0,885	L_5L	0,923	L_5L	0,877	L_5L	0,765	L_5L	0,777	L_5L	0,830
L_24dd	0,901	L_24dd	0,894	L_24dd	0,845	L_24dd	0,762	L_24dd	0,761	L_24dd	0,794
L_24dv	0,862	L_24dv	0,873	L_24dv	0,844	L_24dv	0,676	L_24dv	0,631	L_24dv	0,728
L_7AL	0,914	L_7AL	0,924	L_7AL	0,832	L_7AL	0,734	L_7AL	0,768	L_7AL	0,813
L_SCEF	0,824	L_SCEF	0,906	L_SCEF	0,844	L_SCEF	0,696	L_SCEF	0,717	L_SCEF	0,717
L_6ma	0,856	L_6ma	0,885	L_6ma	0,865	L_6ma	0,752	L_6ma	0,652	L_6ma	0,784
L_7Am	0,906	L_7Am	0,932	L_7Am	0,879	L_7Am	0,775	L_7Am	0,755	L_7Am	0,792
L_7PL	0,919	L_7PL	0,927	L_7PL	0,873	L_7PL	0,713	L_7PL	0,743	L_7PL	0,773
L_7PC	0,894	L_7PC	0,896	L_7PC	0,865	L_7PC	0,757	L_7PC	0,771	L_7PC	0,773
L_LIPv	0,869	L_LIPv	0,907	L_LIPv	0,846	L_LIPv	0,730	L_LIPv	0,714	L_LIPv	0,738
L_VIP	0,907	L_VIP	0,913	L_VIP	0,878	L_VIP	0,770	L_VIP	0,802	L_VIP	0,795
L_MIP	0,896	L_MIP	0,931	L_MIP	0,875	L_MIP	0,755	L_MIP	0,851	L_MIP	0,755
L_1	0,931	L_1	0,927	L_1	0,909	L_1	0,810	L_1	0,796	L_1	0,857
L_2	0,884	L_2	0,921	L_2	0,874	L_2	0,817	L_2	0,853	L_2	0,762
L_3a	0,846	L_3a	0,908	L_3a	0,844	L_3a	0,811	L_3a	0,756	L_3a	0,799
L_6d	0,910	L_6d	0,931	L_6d	0,888	L_6d	0,818	L_6d	0,846	L_6d	0,807
L_6mp	0,901	L_6mp	0,918	L_6mp	0,873	L_6mp	0,745	L_6mp	0,843	L_6mp	0,815

L_6v	0,875	L_6v	0,912	L_6v	0,858	L_6v	0,794	L_6v	0,628	L_6v	0,738
L_p24pr	0,863	L_p24pr	0,895	L_p24pr	0,780	L_p24pr	0,724	L_p24pr	0,718	L_p24pr	0,752
L_33pr	0,905	L_33pr	0,888	L_33pr	0,841	L_33pr	0,621	L_33pr	0,708	L_33pr	0,719
L_a24pr	0,851	L_a24pr	0,861	L_a24pr	0,834	L_a24pr	0,695	L_a24pr	0,709	L_a24pr	0,743
L_p32pr	0,876	L_p32pr	0,896	L_p32pr	0,849	L_p32pr	0,737	L_p32pr	0,738	L_p32pr	0,761
L_a24	0,905	L_a24	0,896	L_a24	0,848	L_a24	0,734	L_a24	0,790	L_a24	0,748
L_d32	0,870	L_d32	0,893	L_d32	0,854	L_d32	0,787	L_d32	0,772	L_d32	0,749
L_8BM	0,874	L_8BM	0,874	L_8BM	0,819	L_8BM	0,679	L_8BM	0,708	L_8BM	0,709
L_p32	0,897	L_p32	0,863	L_p32	0,817	L_p32	0,768	L_p32	0,783	L_p32	0,787
L_10r	0,904	L_10r	0,891	L_10r	0,852	L_10r	0,791	L_10r	0,796	L_10r	0,828
L_47m	0,881	L_47m	0,882	L_47m	0,805	L_47m	0,709	L_47m	0,730	L_47m	0,782
L_8Av	0,930	L_8Av	0,872	L_8Av	0,882	L_8Av	0,730	L_8Av	0,764	L_8Av	0,769
L_8Ad	0,871	L_8Ad	0,871	L_8Ad	0,802	L_8Ad	0,770	L_8Ad	0,663	L_8Ad	0,751
L_9m	0,913	L_9m	0,923	L_9m	0,871	L_9m	0,831	L_9m	0,782	L_9m	0,740
L_8BL	0,910	L_8BL	0,872	L_8BL	0,842	L_8BL	0,800	L_8BL	0,735	L_8BL	0,818
L_9p	0,892	L_9p	0,875	L_9p	0,843	L_9p	0,774	L_9p	0,758	L_9p	0,759
L_10d	0,912	L_10d	0,909	L_10d	0,878	L_10d	0,805	L_10d	0,780	L_10d	0,766
L_8C	0,888	L_8C	0,889	L_8C	0,871	L_8C	0,746	L_8C	0,683	L_8C	0,801
L_44	0,876	L_44	0,870	L_44	0,837	L_44	0,749	L_44	0,751	L_44	0,763
L_45	0,864	L_45	0,898	L_45	0,833	L_45	0,790	L_45	0,651	L_45	0,769
L_47l	0,881	L_47l	0,890	L_47l	0,830	L_47l	0,720	L_47l	0,670	L_47l	0,777
L_a47r	0,900	L_a47r	0,900	L_a47r	0,868	L_a47r	0,740	L_a47r	0,801	L_a47r	0,809
L_6r	0,866	L_6r	0,892	L_6r	0,861	L_6r	0,762	L_6r	0,735	L_6r	0,707
L_IFJa	0,864	L_IFJa	0,907	L_IFJa	0,857	L_IFJa	0,730	L_IFJa	0,689	L_IFJa	0,774
L_IFJp	0,865	L_IFJp	0,907	L_IFJp	0,826	L_IFJp	0,786	L_IFJp	0,767	L_IFJp	0,753
L_IFSp	0,873	L_IFSp	0,888	L_IFSp	0,855	L_IFSp	0,685	L_IFSp	0,777	L_IFSp	0,728
L_IFSa	0,865	L_IFSa	0,915	L_IFSa	0,852	L_IFSa	0,699	L_IFSa	0,760	L_IFSa	0,711
L_p9.46v	0,875	L_p9.46v	0,904	L_p9.46v	0,810	L_p9.46v	0,790	L_p9.46v	0,798	L_p9.46v	0,774
L_46	0,891	L_46	0,887	L_46	0,827	L_46	0,789	L_46	0,725	L_46	0,730
L_a9.46v	0,891	L_a9.46v	0,896	L_a9.46v	0,858	L_a9.46v	0,685	L_a9.46v	0,693	L_a9.46v	0,743

L_9.46d	0,882	L_9.46d	0,906	L_9.46d	0,828	L_9.46d	0,794	L_9.46d	0,765	L_9.46d	0,720
L_9a	0,913	L_9a	0,888	L_9a	0,869	L_9a	0,760	L_9a	0,741	L_9a	0,784
L_10v	0,919	L_10v	0,891	L_10v	0,870	L_10v	0,774	L_10v	0,819	L_10v	0,800
L_a10p	0,905	L_a10p	0,914	L_a10p	0,877	L_a10p	0,740	L_a10p	0,807	L_a10p	0,720
L_10pp	0,918	L_10pp	0,920	L_10pp	0,876	L_10pp	0,704	L_10pp	0,863	L_10pp	0,783
L_11l	0,904	L_11l	0,900	L_11l	0,853	L_11l	0,743	L_11l	0,867	L_11l	0,837
L_13l	0,895	L_13l	0,909	L_13l	0,857	L_13l	0,756	L_13l	0,896	L_13l	0,876
L_OFC	0,908	L_OFC	0,942	L_OFC	0,877	L_OFC	0,685	L_OFC	0,885	L_OFC	0,894
L_47s	0,872	L_47s	0,873	L_47s	0,791	L_47s	0,759	L_47s	0,681	L_47s	0,718
L_LIPd	0,888	L_LIPd	0,903	L_LIPd	0,858	L_LIPd	0,791	L_LIPd	0,772	L_LIPd	0,764
L_6a	0,866	L_6a	0,900	L_6a	0,870	L_6a	0,757	L_6a	0,663	L_6a	0,723
L_i6.8	0,887	L_i6.8	0,876	L_i6.8	0,850	L_i6.8	0,700	L_i6.8	0,751	L_i6.8	0,757
L_s6.8	0,892	L_s6.8	0,889	L_s6.8	0,841	L_s6.8	0,751	L_s6.8	0,721	L_s6.8	0,778
L_43	0,848	L_43	0,892	L_43	0,844	L_43	0,762	L_43	0,746	L_43	0,737
L_OP4	0,890	L_OP4	0,918	L_OP4	0,893	L_OP4	0,761	L_OP4	0,783	L_OP4	0,713
L_OP1	0,857	L_OP1	0,903	L_OP1	0,842	L_OP1	0,700	L_OP1	0,742	L_OP1	0,746
L_OP2.3	0,877	L_OP2.3	0,906	L_OP2.3	0,831	L_OP2.3	0,791	L_OP2.3	0,730	L_OP2.3	0,707
L_52	0,843	L_52	0,888	L_52	0,811	L_52	0,719	L_52	0,746	L_52	0,732
L_RI	0,841	L_RI	0,920	L_RI	0,861	L_RI	0,768	L_RI	0,675	L_RI	0,775
L_PFcm	0,850	L_PFcm	0,888	L_PFcm	0,873	L_PFcm	0,724	L_PFcm	0,788	L_PFcm	0,799
L_PoI2	0,843	L_PoI2	0,867	L_PoI2	0,805	L_PoI2	0,722	L_PoI2	0,713	L_PoI2	0,651
L_TA2	0,872	L_TA2	0,894	L_TA2	0,832	L_TA2	0,640	L_TA2	0,664	L_TA2	0,670
L_FOP4	0,854	L_FOP4	0,884	L_FOP4	0,808	L_FOP4	0,767	L_FOP4	0,767	L_FOP4	0,740
L_MI	0,857	L_MI	0,865	L_MI	0,859	L_MI	0,724	L_MI	0,770	L_MI	0,704
L_Pir	0,905	L_Pir	0,843	L_Pir	0,722	L_Pir	0,544	L_Pir	0,679	L_Pir	0,662
L_AVI	0,875	L_AVI	0,873	L_AVI	0,800	L_AVI	0,706	L_AVI	0,690	L_AVI	0,725
L_AAIC	0,881	L_AAIC	0,837	L_AAIC	0,801	L_AAIC	0,678	L_AAIC	0,693	L_AAIC	0,634
L_FOP1	0,860	L_FOP1	0,863	L_FOP1	0,818	L_FOP1	0,718	L_FOP1	0,652	L_FOP1	0,734
L_FOP3	0,888	L_FOP3	0,872	L_FOP3	0,815	L_FOP3	0,719	L_FOP3	0,744	L_FOP3	0,750
L_FOP2	0,885	L_FOP2	0,894	L_FOP2	0,812	L_FOP2	0,772	L_FOP2	0,660	L_FOP2	0,748

L_PFt	0,884	L_PFt	0,898	L_PFt	0,891	L_PFt	0,819	L_PFt	0,797	L_PFt	0,737
L_AIP	0,863	L_AIP	0,895	L_AIP	0,869	L_AIP	0,782	L_AIP	0,746	L_AIP	0,766
L_EC	0,868	L_EC	0,831	L_EC	0,651	L_EC	0,492	L_EC	0,605	L_EC	0,727
L_PreS	0,857	L_PreS	0,861	L_PreS	0,832	L_PreS	0,694	L_PreS	0,588	L_PreS	0,634
L_H	0,827	L_H	0,956	L_H	0,896	L_H	0,894	L_H	0,884	L_H	0,977
L_ProS	0,876	L_ProS	0,920	L_ProS	0,892	L_ProS	0,792	L_ProS	0,808	L_ProS	0,809
L_PeEc	0,871	L_PeEc	0,878	L_PeEc	0,835	L_PeEc	0,679	L_PeEc	0,785	L_PeEc	0,777
L_STGa	0,859	L_STGa	0,877	L_STGa	0,866	L_STGa	0,759	L_STGa	0,639	L_STGa	0,725
L_PBelt	0,885	L_PBelt	0,928	L_PBelt	0,898	L_PBelt	0,791	L_PBelt	0,805	L_PBelt	0,792
L_A5	0,875	L_A5	0,898	L_A5	0,873	L_A5	0,787	L_A5	0,734	L_A5	0,753
L_PHA1	0,821	L_PHA1	0,889	L_PHA1	0,869	L_PHA1	0,700	L_PHA1	0,722	L_PHA1	0,720
L_PHA3	0,849	L_PHA3	0,904	L_PHA3	0,869	L_PHA3	0,686	L_PHA3	0,770	L_PHA3	0,724
L_STSda	0,850	L_STSda	0,898	L_STSda	0,895	L_STSda	0,837	L_STSda	0,708	L_STSda	0,791
L_STSdp	0,857	L_STSdp	0,895	L_STSdp	0,906	L_STSdp	0,862	L_STSdp	0,784	L_STSdp	0,764
L_STSvp	0,880	L_STSvp	0,904	L_STSvp	0,898	L_STSvp	0,765	L_STSvp	0,755	L_STSvp	0,748
L_TGd	0,886	L_TGd	0,888	L_TGd	0,836	L_TGd	0,736	L_TGd	0,787	L_TGd	0,752
L_TE1a	0,880	L_TE1a	0,888	L_TE1a	0,841	L_TE1a	0,787	L_TE1a	0,677	L_TE1a	0,743
L_TE1p	0,919	L_TE1p	0,879	L_TE1p	0,843	L_TE1p	0,758	L_TE1p	0,736	L_TE1p	0,723
L_TE2a	0,888	L_TE2a	0,858	L_TE2a	0,798	L_TE2a	0,697	L_TE2a	0,755	L_TE2a	0,732
L_TF	0,864	L_TF	0,872	L_TF	0,776	L_TF	0,672	L_TF	0,774	L_TF	0,719
L_TE2p	0,860	L_TE2p	0,852	L_TE2p	0,845	L_TE2p	0,734	L_TE2p	0,698	L_TE2p	0,719
L_PHT	0,901	L_PHT	0,871	L_PHT	0,827	L_PHT	0,738	L_PHT	0,747	L_PHT	0,654
L_PH	0,865	L_PH	0,898	L_PH	0,860	L_PH	0,762	L_PH	0,755	L_PH	0,772
L_TPOJ1	0,872	L_TPOJ1	0,893	L_TPOJ1	0,897	L_TPOJ1	0,784	L_TPOJ1	0,766	L_TPOJ1	0,720
L_TPOJ2	0,878	L_TPOJ2	0,878	L_TPOJ2	0,902	L_TPOJ2	0,784	L_TPOJ2	0,686	L_TPOJ2	0,721
L_TPOJ3	0,863	L_TPOJ3	0,903	L_TPOJ3	0,864	L_TPOJ3	0,747	L_TPOJ3	0,704	L_TPOJ3	0,718
L_DVT	0,878	L_DVT	0,909	L_DVT	0,869	L_DVT	0,713	L_DVT	0,807	L_DVT	0,791
L_PGp	0,896	L_PGp	0,907	L_PGp	0,852	L_PGp	0,753	L_PGp	0,798	L_PGp	0,755
L_IP2	0,866	L_IP2	0,907	L_IP2	0,886	L_IP2	0,745	L_IP2	0,802	L_IP2	0,774
L_IP1	0,890	L_IP1	0,904	L_IP1	0,852	L_IP1	0,772	L_IP1	0,768	L_IP1	0,775

L_IP0	0,859	L_IP0	0,918	L_IP0	0,901	L_IP0	0,741	L_IP0	0,815	L_IP0	0,820
L_PFop	0,885	L_PFop	0,918	L_PFop	0,833	L_PFop	0,779	L_PFop	0,727	L_PFop	0,751
L_PF	0,909	L_PF	0,903	L_PF	0,837	L_PF	0,757	L_PF	0,714	L_PF	0,703
L_PFm	0,916	L_PFm	0,855	L_PFm	0,848	L_PFm	0,725	L_PFm	0,794	L_PFm	0,769
L_PGi	0,914	L_PGi	0,858	L_PGi	0,852	L_PGi	0,789	L_PGi	0,760	L_PGi	0,718
L_PGs	0,912	L_PGs	0,904	L_PGs	0,871	L_PGs	0,784	L_PGs	0,726	L_PGs	0,769
L_V6A	0,916	L_V6A	0,904	L_V6A	0,855	L_V6A	0,791	L_V6A	0,754	L_V6A	0,806
L_VMV1	0,867	L_VMV1	0,903	L_VMV1	0,814	L_VMV1	0,773	L_VMV1	0,778	L_VMV1	0,766
L_VMV3	0,883	L_VMV3	0,927	L_VMV3	0,882	L_VMV3	0,803	L_VMV3	0,780	L_VMV3	0,818
L_PHA2	0,827	L_PHA2	0,908	L_PHA2	0,865	L_PHA2	0,683	L_PHA2	0,705	L_PHA2	0,700
L_V4t	0,909	L_V4t	0,924	L_V4t	0,903	L_V4t	0,788	L_V4t	0,789	L_V4t	0,837
L_FST	0,869	L_FST	0,915	L_FST	0,875	L_FST	0,735	L_FST	0,760	L_FST	0,790
L_V3CD	0,895	L_V3CD	0,936	L_V3CD	0,905	L_V3CD	0,824	L_V3CD	0,769	L_V3CD	0,851
L_LO3	0,900	L_LO3	0,920	L_LO3	0,907	L_LO3	0,788	L_LO3	0,797	L_LO3	0,806
L_VMV2	0,872	L_VMV2	0,919	L_VMV2	0,865	L_VMV2	0,795	L_VMV2	0,787	L_VMV2	0,801
L_31pd	0,886	L_31pd	0,904	L_31pd	0,852	L_31pd	0,829	L_31pd	0,766	L_31pd	0,751
L_31a	0,850	L_31a	0,882	L_31a	0,838	L_31a	0,747	L_31a	0,791	L_31a	0,752
L_VVC	0,866	L_VVC	0,877	L_VVC	0,860	L_VVC	0,786	L_VVC	0,746	L_VVC	0,794
L_25	0,895	L_25	0,926	L_25	0,869	L_25	0,720	L_25	0,829	L_25	0,885
L_s32	0,886	L_s32	0,875	L_s32	0,878	L_s32	0,732	L_s32	0,802	L_s32	0,865
L_pOFC	0,910	L_pOFC	0,939	L_pOFC	0,845	L_pOFC	0,662	L_pOFC	0,842	L_pOFC	0,869
L_PoI1	0,845	L_PoI1	0,858	L_PoI1	0,813	L_PoI1	0,661	L_PoI1	0,712	L_PoI1	0,767
L_Ig	0,865	L_Ig	0,908	L_Ig	0,866	L_Ig	0,761	L_Ig	0,724	L_Ig	0,767
L_FOP5	0,879	L_FOP5	0,879	L_FOP5	0,816	L_FOP5	0,759	L_FOP5	0,773	L_FOP5	0,723
L_p10p	0,897	L_p10p	0,905	L_p10p	0,850	L_p10p	0,752	L_p10p	0,739	L_p10p	0,742
L_p47r	0,878	L_p47r	0,881	L_p47r	0,839	L_p47r	0,752	L_p47r	0,798	L_p47r	0,691
L_TGv	0,881	L_TGv	0,877	L_TGv	0,816	L_TGv	0,662	L_TGv	0,749	L_TGv	0,764
L_MBelt	0,856	L_MBelt	0,932	L_MBelt	0,887	L_MBelt	0,735	L_MBelt	0,720	L_MBelt	0,788
L_LBelt	0,859	L_LBelt	0,930	L_LBelt	0,902	L_LBelt	0,777	L_LBelt	0,759	L_LBelt	0,791
L_A4	0,894	L_A4	0,925	L_A4	0,885	L_A4	0,775	L_A4	0,777	L_A4	0,802

L_STSva	0,859	L_STSva	0,896	L_STSva	0,855	L_STSva	0,777	L_STSva	0,747	L_STSva	0,808
L_TE1m	0,913	L_TE1m	0,865	L_TE1m	0,812	L_TE1m	0,739	L_TE1m	0,703	L_TE1m	0,739
L_PI	0,876	L_PI	0,797	L_PI	0,744	L_PI	0,588	L_PI	0,679	L_PI	0,680
L_a32pr	0,845	L_a32pr	0,871	L_a32pr	0,786	L_a32pr	0,704	L_a32pr	0,775	L_a32pr	0,725
L_p24	0,887	L_p24	0,885	L_p24	0,822	L_p24	0,711	L_p24	0,726	L_p24	0,699
R_V1	0,915	R_V1	0,930	R_V1	0,921	R_V1	0,856	R_V1	0,859	R_V1	0,864
R_MST	0,866	R_MST	0,929	R_MST	0,924	R_MST	0,767	R_MST	0,807	R_MST	0,771
R_V6	0,867	R_V6	0,908	R_V6	0,860	R_V6	0,799	R_V6	0,743	R_V6	0,761
R_V2	0,909	R_V2	0,921	R_V2	0,907	R_V2	0,856	R_V2	0,831	R_V2	0,854
R_V3	0,935	R_V3	0,929	R_V3	0,912	R_V3	0,835	R_V3	0,809	R_V3	0,869
R_V4	0,936	R_V4	0,948	R_V4	0,897	R_V4	0,833	R_V4	0,799	R_V4	0,878
R_V8	0,889	R_V8	0,929	R_V8	0,852	R_V8	0,842	R_V8	0,785	R_V8	0,831
R_4	0,900	R_4	0,922	R_4	0,888	R_4	0,836	R_4	0,849	R_4	0,842
R_3b	0,897	R_3b	0,915	R_3b	0,878	R_3b	0,870	R_3b	0,793	R_3b	0,850
R_FEF	0,847	R_FEF	0,872	R_FEF	0,828	R_FEF	0,751	R_FEF	0,678	R_FEF	0,666
R_PEF	0,868	R_PEF	0,892	R_PEF	0,821	R_PEF	0,722	R_PEF	0,717	R_PEF	0,684
R_55b	0,857	R_55b	0,879	R_55b	0,804	R_55b	0,734	R_55b	0,695	R_55b	0,706
R_V3A	0,936	R_V3A	0,917	R_V3A	0,915	R_V3A	0,843	R_V3A	0,835	R_V3A	0,875
R_RSC	0,839	R_RSC	0,902	R_RSC	0,888	R_RSC	0,716	R_RSC	0,709	R_RSC	0,732
R_POS2	0,879	R_POS2	0,907	R_POS2	0,844	R_POS2	0,677	R_POS2	0,760	R_POS2	0,764
R_V7	0,911	R_V7	0,939	R_V7	0,888	R_V7	0,820	R_V7	0,826	R_V7	0,828
R_IPS1	0,891	R_IPS1	0,915	R_IPS1	0,884	R_IPS1	0,781	R_IPS1	0,844	R_IPS1	0,787
R_FFC	0,892	R_FFC	0,908	R_FFC	0,871	R_FFC	0,757	R_FFC	0,784	R_FFC	0,801
R_V3B	0,909	R_V3B	0,919	R_V3B	0,862	R_V3B	0,821	R_V3B	0,728	R_V3B	0,816
R_LO1	0,916	R_LO1	0,938	R_LO1	0,921	R_LO1	0,842	R_LO1	0,848	R_LO1	0,838
R_LO2	0,929	R_LO2	0,952	R_LO2	0,899	R_LO2	0,815	R_LO2	0,848	R_LO2	0,850
R_PIT	0,899	R_PIT	0,938	R_PIT	0,899	R_PIT	0,814	R_PIT	0,802	R_PIT	0,834
R_MT	0,893	R_MT	0,929	R_MT	0,928	R_MT	0,758	R_MT	0,851	R_MT	0,817
R_A1	0,874	R_A1	0,928	R_A1	0,904	R_A1	0,783	R_A1	0,725	R_A1	0,802
R_PSL	0,882	R_PSL	0,877	R_PSL	0,803	R_PSL	0,715	R_PSL	0,754	R_PSL	0,772

R_SFL	0,866	R_SFL	0,877	R_SFL	0,809	R_SFL	0,685	R_SFL	0,685	R_SFL	0,789
R_PCV	0,854	R_PCV	0,864	R_PCV	0,869	R_PCV	0,715	R_PCV	0,739	R_PCV	0,790
R_STV	0,857	R_STV	0,918	R_STV	0,866	R_STV	0,782	R_STV	0,739	R_STV	0,779
R_7Pm	0,872	R_7Pm	0,891	R_7Pm	0,881	R_7Pm	0,735	R_7Pm	0,740	R_7Pm	0,757
R_7m	0,894	R_7m	0,920	R_7m	0,919	R_7m	0,835	R_7m	0,735	R_7m	0,825
R_POS1	0,862	R_POS1	0,852	R_POS1	0,805	R_POS1	0,754	R_POS1	0,647	R_POS1	0,710
R_23d	0,878	R_23d	0,880	R_23d	0,813	R_23d	0,720	R_23d	0,665	R_23d	0,698
R_v23ab	0,857	R_v23ab	0,893	R_v23ab	0,853	R_v23ab	0,805	R_v23ab	0,737	R_v23ab	0,719
R_d23ab	0,862	R_d23ab	0,900	R_d23ab	0,880	R_d23ab	0,785	R_d23ab	0,763	R_d23ab	0,770
R_31pv	0,857	R_31pv	0,887	R_31pv	0,854	R_31pv	0,782	R_31pv	0,789	R_31pv	0,784
R_5m	0,906	R_5m	0,934	R_5m	0,900	R_5m	0,779	R_5m	0,800	R_5m	0,846
R_5mv	0,882	R_5mv	0,887	R_5mv	0,868	R_5mv	0,688	R_5mv	0,716	R_5mv	0,720
R_23c	0,821	R_23c	0,881	R_23c	0,825	R_23c	0,758	R_23c	0,662	R_23c	0,681
R_5L	0,908	R_5L	0,940	R_5L	0,903	R_5L	0,790	R_5L	0,814	R_5L	0,816
R_24dd	0,894	R_24dd	0,904	R_24dd	0,863	R_24dd	0,813	R_24dd	0,756	R_24dd	0,765
R_24dv	0,882	R_24dv	0,897	R_24dv	0,828	R_24dv	0,722	R_24dv	0,688	R_24dv	0,717
R_7AL	0,899	R_7AL	0,906	R_7AL	0,845	R_7AL	0,756	R_7AL	0,814	R_7AL	0,773
R_SCEF	0,853	R_SCEF	0,897	R_SCEF	0,850	R_SCEF	0,736	R_SCEF	0,692	R_SCEF	0,734
R_6ma	0,878	R_6ma	0,895	R_6ma	0,848	R_6ma	0,761	R_6ma	0,693	R_6ma	0,797
R_7Am	0,914	R_7Am	0,905	R_7Am	0,889	R_7Am	0,718	R_7Am	0,762	R_7Am	0,802
R_7PL	0,894	R_7PL	0,910	R_7PL	0,889	R_7PL	0,762	R_7PL	0,783	R_7PL	0,775
R_7PC	0,909	R_7PC	0,909	R_7PC	0,876	R_7PC	0,820	R_7PC	0,764	R_7PC	0,784
R_LIPv	0,895	R_LIPv	0,910	R_LIPv	0,882	R_LIPv	0,793	R_LIPv	0,781	R_LIPv	0,790
R_VIP	0,899	R_VIP	0,893	R_VIP	0,842	R_VIP	0,776	R_VIP	0,810	R_VIP	0,746
R_MIP	0,891	R_MIP	0,919	R_MIP	0,890	R_MIP	0,806	R_MIP	0,832	R_MIP	0,801
R_1	0,930	R_1	0,917	R_1	0,898	R_1	0,847	R_1	0,816	R_1	0,838
R_2	0,890	R_2	0,932	R_2	0,897	R_2	0,817	R_2	0,838	R_2	0,764
R_3a	0,866	R_3a	0,904	R_3a	0,848	R_3a	0,822	R_3a	0,791	R_3a	0,824
R_6d	0,897	R_6d	0,936	R_6d	0,891	R_6d	0,838	R_6d	0,783	R_6d	0,850
R_6mp	0,881	R_6mp	0,925	R_6mp	0,862	R_6mp	0,802	R_6mp	0,821	R_6mp	0,810

R_6v	0,861	R_6v	0,925	R_6v	0,866	R_6v	0,753	R_6v	0,722	R_6v	0,713
R_p24pr	0,888	R_p24pr	0,877	R_p24pr	0,801	R_p24pr	0,740	R_p24pr	0,670	R_p24pr	0,753
R_33pr	0,917	R_33pr	0,890	R_33pr	0,809	R_33pr	0,644	R_33pr	0,692	R_33pr	0,723
R_a24pr	0,898	R_a24pr	0,882	R_a24pr	0,864	R_a24pr	0,711	R_a24pr	0,694	R_a24pr	0,717
R_p32pr	0,847	R_p32pr	0,914	R_p32pr	0,841	R_p32pr	0,784	R_p32pr	0,772	R_p32pr	0,713
R_a24	0,901	R_a24	0,875	R_a24	0,842	R_a24	0,721	R_a24	0,759	R_a24	0,769
R_d32	0,852	R_d32	0,881	R_d32	0,840	R_d32	0,661	R_d32	0,723	R_d32	0,740
R_8BM	0,870	R_8BM	0,877	R_8BM	0,793	R_8BM	0,742	R_8BM	0,746	R_8BM	0,735
R_p32	0,897	R_p32	0,902	R_p32	0,847	R_p32	0,687	R_p32	0,807	R_p32	0,785
R_10r	0,889	R_10r	0,904	R_10r	0,855	R_10r	0,784	R_10r	0,812	R_10r	0,827
R_47m	0,877	R_47m	0,831	R_47m	0,824	R_47m	0,722	R_47m	0,786	R_47m	0,783
R_8Av	0,912	R_8Av	0,895	R_8Av	0,857	R_8Av	0,748	R_8Av	0,796	R_8Av	0,750
R_8Ad	0,862	R_8Ad	0,889	R_8Ad	0,802	R_8Ad	0,787	R_8Ad	0,646	R_8Ad	0,686
R_9m	0,920	R_9m	0,920	R_9m	0,856	R_9m	0,836	R_9m	0,848	R_9m	0,802
R_8BL	0,907	R_8BL	0,873	R_8BL	0,832	R_8BL	0,745	R_8BL	0,731	R_8BL	0,727
R_9p	0,885	R_9p	0,874	R_9p	0,825	R_9p	0,748	R_9p	0,720	R_9p	0,718
R_10d	0,922	R_10d	0,897	R_10d	0,863	R_10d	0,747	R_10d	0,755	R_10d	0,772
R_8C	0,893	R_8C	0,899	R_8C	0,864	R_8C	0,761	R_8C	0,748	R_8C	0,745
R_44	0,870	R_44	0,902	R_44	0,820	R_44	0,663	R_44	0,724	R_44	0,688
R_45	0,851	R_45	0,859	R_45	0,836	R_45	0,790	R_45	0,723	R_45	0,698
R_47l	0,862	R_47l	0,840	R_47l	0,794	R_47l	0,718	R_47l	0,682	R_47l	0,714
R_a47r	0,916	R_a47r	0,905	R_a47r	0,850	R_a47r	0,758	R_a47r	0,765	R_a47r	0,796
R_6r	0,867	R_6r	0,878	R_6r	0,817	R_6r	0,696	R_6r	0,782	R_6r	0,781
R_IFJa	0,863	R_IFJa	0,893	R_IFJa	0,867	R_IFJa	0,738	R_IFJa	0,733	R_IFJa	0,707
R_IFJp	0,855	R_IFJp	0,910	R_IFJp	0,848	R_IFJp	0,718	R_IFJp	0,775	R_IFJp	0,744
R_IFSp	0,863	R_IFSp	0,900	R_IFSp	0,838	R_IFSp	0,703	R_IFSp	0,719	R_IFSp	0,744
R_IFSa	0,843	R_IFSa	0,880	R_IFSa	0,838	R_IFSa	0,670	R_IFSa	0,764	R_IFSa	0,691
R_p9.46v	0,915	R_p9.46v	0,895	R_p9.46v	0,864	R_p9.46v	0,808	R_p9.46v	0,794	R_p9.46v	0,728
R_46	0,901	R_46	0,883	R_46	0,830	R_46	0,768	R_46	0,704	R_46	0,708
R_a9.46v	0,920	R_a9.46v	0,877	R_a9.46v	0,833	R_a9.46v	0,710	R_a9.46v	0,787	R_a9.46v	0,769

R_9.46d	0,909	R_9.46d	0,907	R_9.46d	0,847	R_9.46d	0,753	R_9.46d	0,782	R_9.46d	0,752
R_9a	0,910	R_9a	0,897	R_9a	0,839	R_9a	0,810	R_9a	0,770	R_9a	0,734
R_10v	0,902	R_10v	0,905	R_10v	0,857	R_10v	0,761	R_10v	0,791	R_10v	0,785
R_a10p	0,929	R_a10p	0,919	R_a10p	0,853	R_a10p	0,729	R_a10p	0,773	R_a10p	0,762
R_10pp	0,914	R_10pp	0,926	R_10pp	0,870	R_10pp	0,758	R_10pp	0,865	R_10pp	0,786
R_11l	0,897	R_11l	0,908	R_11l	0,858	R_11l	0,753	R_11l	0,804	R_11l	0,849
R_13l	0,911	R_13l	0,943	R_13l	0,867	R_13l	0,746	R_13l	0,874	R_13l	0,870
R_OFC	0,918	R_OFC	0,938	R_OFC	0,896	R_OFC	0,691	R_OFC	0,843	R_OFC	0,889
R_47s	0,843	R_47s	0,829	R_47s	0,793	R_47s	0,687	R_47s	0,711	R_47s	0,694
R_LIPd	0,859	R_LIPd	0,905	R_LIPd	0,879	R_LIPd	0,786	R_LIPd	0,799	R_LIPd	0,821
R_6a	0,862	R_6a	0,889	R_6a	0,847	R_6a	0,767	R_6a	0,614	R_6a	0,710
R_i6.8	0,859	R_i6.8	0,896	R_i6.8	0,876	R_i6.8	0,757	R_i6.8	0,764	R_i6.8	0,798
R_s6.8	0,870	R_s6.8	0,875	R_s6.8	0,836	R_s6.8	0,733	R_s6.8	0,745	R_s6.8	0,746
R_43	0,835	R_43	0,905	R_43	0,865	R_43	0,757	R_43	0,737	R_43	0,795
R_OP4	0,884	R_OP4	0,903	R_OP4	0,862	R_OP4	0,801	R_OP4	0,792	R_OP4	0,802
R_OP1	0,877	R_OP1	0,915	R_OP1	0,866	R_OP1	0,785	R_OP1	0,778	R_OP1	0,828
R_OP2.3	0,868	R_OP2.3	0,900	R_OP2.3	0,853	R_OP2.3	0,670	R_OP2.3	0,724	R_OP2.3	0,825
R_52	0,862	R_52	0,903	R_52	0,843	R_52	0,650	R_52	0,764	R_52	0,830
R_RI	0,838	R_RI	0,892	R_RI	0,861	R_RI	0,761	R_RI	0,668	R_RI	0,778
R_PFcm	0,870	R_PFcm	0,888	R_PFcm	0,825	R_PFcm	0,690	R_PFcm	0,698	R_PFcm	0,781
R_PoI2	0,817	R_PoI2	0,850	R_PoI2	0,798	R_PoI2	0,689	R_PoI2	0,648	R_PoI2	0,799
R_TA2	0,862	R_TA2	0,899	R_TA2	0,825	R_TA2	0,708	R_TA2	0,707	R_TA2	0,738
R_FOP4	0,827	R_FOP4	0,917	R_FOP4	0,827	R_FOP4	0,791	R_FOP4	0,751	R_FOP4	0,747
R_MI	0,846	R_MI	0,900	R_MI	0,816	R_MI	0,708	R_MI	0,778	R_MI	0,722
R_Pir	0,878	R_Pir	0,814	R_Pir	0,766	R_Pir	0,529	R_Pir	0,577	R_Pir	0,717
R_AVI	0,825	R_AVI	0,890	R_AVI	0,816	R_AVI	0,673	R_AVI	0,721	R_AVI	0,605
R_AAIC	0,832	R_AAIC	0,869	R_AAIC	0,797	R_AAIC	0,694	R_AAIC	0,725	R_AAIC	0,616
R_FOP1	0,863	R_FOP1	0,868	R_FOP1	0,804	R_FOP1	0,773	R_FOP1	0,719	R_FOP1	0,778
R_FOP3	0,846	R_FOP3	0,894	R_FOP3	0,790	R_FOP3	0,781	R_FOP3	0,697	R_FOP3	0,740
R_FOP2	0,887	R_FOP2	0,898	R_FOP2	0,825	R_FOP2	0,718	R_FOP2	0,630	R_FOP2	0,804

R_PFt	0,879	R_PFt	0,891	R_PFt	0,881	R_PFt	0,787	R_PFt	0,748	R_PFt	0,796
R_AIP	0,885	R_AIP	0,889	R_AIP	0,851	R_AIP	0,786	R_AIP	0,720	R_AIP	0,743
R_EC	0,848	R_EC	0,822	R_EC	0,675	R_EC	0,565	R_EC	0,595	R_EC	0,646
R_PreS	0,862	R_PreS	0,862	R_PreS	0,839	R_PreS	0,686	R_PreS	0,696	R_PreS	0,699
R_H	0,000	R_H	0,926	R_H	0,895	R_H	0,889	R_H	0,905	R_H	0,943
R_ProS	0,858	R_ProS	0,885	R_ProS	0,881	R_ProS	0,779	R_ProS	0,785	R_ProS	0,718
R_PeEc	0,892	R_PeEc	0,870	R_PeEc	0,803	R_PeEc	0,567	R_PeEc	0,731	R_PeEc	0,741
R_STGa	0,853	R_STGa	0,862	R_STGa	0,851	R_STGa	0,736	R_STGa	0,663	R_STGa	0,738
R_PBelt	0,865	R_PBelt	0,942	R_PBelt	0,896	R_PBelt	0,801	R_PBelt	0,682	R_PBelt	0,816
R_A5	0,859	R_A5	0,899	R_A5	0,912	R_A5	0,812	R_A5	0,689	R_A5	0,750
R_PHA1	0,868	R_PHA1	0,908	R_PHA1	0,839	R_PHA1	0,700	R_PHA1	0,768	R_PHA1	0,780
R_PHA3	0,866	R_PHA3	0,896	R_PHA3	0,854	R_PHA3	0,660	R_PHA3	0,799	R_PHA3	0,734
R_STSda	0,868	R_STSda	0,873	R_STSda	0,871	R_STSda	0,778	R_STSda	0,655	R_STSda	0,742
R_STSdp	0,868	R_STSdp	0,916	R_STSdp	0,880	R_STSdp	0,823	R_STSdp	0,754	R_STSdp	0,767
R_STSvp	0,856	R_STSvp	0,891	R_STSvp	0,890	R_STSvp	0,763	R_STSvp	0,777	R_STSvp	0,747
R_TGd	0,858	R_TGd	0,914	R_TGd	0,823	R_TGd	0,709	R_TGd	0,783	R_TGd	0,738
R_TE1a	0,910	R_TE1a	0,866	R_TE1a	0,825	R_TE1a	0,734	R_TE1a	0,600	R_TE1a	0,737
R_TE1p	0,889	R_TE1p	0,865	R_TE1p	0,844	R_TE1p	0,782	R_TE1p	0,731	R_TE1p	0,738
R_TE2a	0,868	R_TE2a	0,869	R_TE2a	0,800	R_TE2a	0,674	R_TE2a	0,728	R_TE2a	0,747
R_TF	0,882	R_TF	0,849	R_TF	0,836	R_TF	0,644	R_TF	0,735	R_TF	0,739
R_TE2p	0,856	R_TE2p	0,878	R_TE2p	0,882	R_TE2p	0,711	R_TE2p	0,735	R_TE2p	0,721
R_PHT	0,896	R_PHT	0,888	R_PHT	0,862	R_PHT	0,757	R_PHT	0,736	R_PHT	0,738
R_PH	0,892	R_PH	0,877	R_PH	0,866	R_PH	0,766	R_PH	0,772	R_PH	0,789
R_TPOJ1	0,855	R_TPOJ1	0,919	R_TPOJ1	0,874	R_TPOJ1	0,795	R_TPOJ1	0,711	R_TPOJ1	0,688
R_TPOJ2	0,884	R_TPOJ2	0,906	R_TPOJ2	0,913	R_TPOJ2	0,731	R_TPOJ2	0,762	R_TPOJ2	0,704
R_TPOJ3	0,867	R_TPOJ3	0,902	R_TPOJ3	0,893	R_TPOJ3	0,680	R_TPOJ3	0,760	R_TPOJ3	0,716
R_DVT	0,865	R_DVT	0,923	R_DVT	0,841	R_DVT	0,677	R_DVT	0,703	R_DVT	0,758
R_PGp	0,908	R_PGp	0,926	R_PGp	0,850	R_PGp	0,765	R_PGp	0,722	R_PGp	0,752
R_IP2	0,882	R_IP2	0,887	R_IP2	0,854	R_IP2	0,762	R_IP2	0,794	R_IP2	0,813
R_IP1	0,901	R_IP1	0,923	R_IP1	0,858	R_IP1	0,788	R_IP1	0,773	R_IP1	0,764

R_IP0	0,877	R_IP0	0,922	R_IP0	0,867	R_IP0	0,762	R_IP0	0,772	R_IP0	0,768
R_PFop	0,897	R_PFop	0,918	R_PFop	0,854	R_PFop	0,714	R_PFop	0,634	R_PFop	0,773
R_PF	0,908	R_PF	0,915	R_PF	0,853	R_PF	0,772	R_PF	0,781	R_PF	0,783
R_PFm	0,916	R_PFm	0,896	R_PFm	0,854	R_PFm	0,766	R_PFm	0,806	R_PFm	0,761
R_PGi	0,910	R_PGi	0,909	R_PGi	0,862	R_PGi	0,808	R_PGi	0,730	R_PGi	0,814
R_PGs	0,922	R_PGs	0,917	R_PGs	0,838	R_PGs	0,752	R_PGs	0,769	R_PGs	0,731
R_V6A	0,911	R_V6A	0,925	R_V6A	0,848	R_V6A	0,790	R_V6A	0,779	R_V6A	0,789
R_VMV1	0,879	R_VMV1	0,913	R_VMV1	0,834	R_VMV1	0,761	R_VMV1	0,786	R_VMV1	0,802
R_VMV3	0,874	R_VMV3	0,932	R_VMV3	0,886	R_VMV3	0,802	R_VMV3	0,700	R_VMV3	0,826
R_PHA2	0,868	R_PHA2	0,890	R_PHA2	0,841	R_PHA2	0,693	R_PHA2	0,710	R_PHA2	0,743
R_V4t	0,902	R_V4t	0,930	R_V4t	0,880	R_V4t	0,813	R_V4t	0,799	R_V4t	0,868
R_FST	0,889	R_FST	0,926	R_FST	0,896	R_FST	0,758	R_FST	0,796	R_FST	0,764
R_V3CD	0,915	R_V3CD	0,936	R_V3CD	0,885	R_V3CD	0,838	R_V3CD	0,748	R_V3CD	0,833
R_LO3	0,911	R_LO3	0,922	R_LO3	0,866	R_LO3	0,787	R_LO3	0,787	R_LO3	0,829
R_VMV2	0,862	R_VMV2	0,913	R_VMV2	0,858	R_VMV2	0,794	R_VMV2	0,752	R_VMV2	0,818
R_31pd	0,877	R_31pd	0,896	R_31pd	0,882	R_31pd	0,755	R_31pd	0,733	R_31pd	0,722
R_31a	0,863	R_31a	0,873	R_31a	0,837	R_31a	0,703	R_31a	0,797	R_31a	0,734
R_VVC	0,847	R_VVC	0,912	R_VVC	0,837	R_VVC	0,801	R_VVC	0,786	R_VVC	0,781
R_25	0,911	R_25	0,921	R_25	0,882	R_25	0,723	R_25	0,791	R_25	0,865
R_s32	0,900	R_s32	0,876	R_s32	0,887	R_s32	0,758	R_s32	0,825	R_s32	0,884
R_pOFC	0,891	R_pOFC	0,923	R_pOFC	0,849	R_pOFC	0,595	R_pOFC	0,854	R_pOFC	0,842
R_PoI1	0,845	R_PoI1	0,881	R_PoI1	0,825	R_PoI1	0,692	R_PoI1	0,734	R_PoI1	0,847
R_Ig	0,874	R_Ig	0,900	R_Ig	0,835	R_Ig	0,803	R_Ig	0,765	R_Ig	0,843
R_FOP5	0,856	R_FOP5	0,886	R_FOP5	0,824	R_FOP5	0,735	R_FOP5	0,694	R_FOP5	0,701
R_p10p	0,906	R_p10p	0,923	R_p10p	0,852	R_p10p	0,736	R_p10p	0,796	R_p10p	0,746
R_p47r	0,878	R_p47r	0,879	R_p47r	0,827	R_p47r	0,703	R_p47r	0,746	R_p47r	0,750
R_TGv	0,871	R_TGv	0,887	R_TGv	0,780	R_TGv	0,640	R_TGv	0,790	R_TGv	0,784
R_MBelt	0,864	R_MBelt	0,933	R_MBelt	0,879	R_MBelt	0,781	R_MBelt	0,758	R_MBelt	0,804
R_LBelt	0,862	R_LBelt	0,939	R_LBelt	0,911	R_LBelt	0,792	R_LBelt	0,746	R_LBelt	0,812
R_A4	0,886	R_A4	0,930	R_A4	0,902	R_A4	0,733	R_A4	0,757	R_A4	0,747

R_STSva	0,856	R_STSva	0,865	R_STSva	0,862	R_STSva	0,703	R_STSva	0,673	R_STSva	0,742
R_TE1m	0,892	R_TE1m	0,878	R_TE1m	0,834	R_TE1m	0,718	R_TE1m	0,686	R_TE1m	0,672
R_PI	0,885	R_PI	0,852	R_PI	0,788	R_PI	0,543	R_PI	0,681	R_PI	0,786
R_a32pr	0,894	R_a32pr	0,895	R_a32pr	0,775	R_a32pr	0,718	R_a32pr	0,768	R_a32pr	0,752
R_p24	0,900	R_p24	0,874	R_p24	0,840	R_p24	0,686	R_p24	0,769	R_p24	0,693
L_accumbens	0,906	L_accumbens	0,919	L_accumbens	0,836	L_accumbens	0,578	L_accumbens	0,838	L_accumbens	0,831
L_amygdala	0,853	L_amygdala	0,845	L_amygdala	0,759	L_amygdala	0,671	L_amygdala	0,724	L_amygdala	0,650
L_caudate	0,857	L_caudate	0,823	L_caudate	0,821	L_caudate	0,578	L_caudate	0,695	L_caudate	0,637
L_hippocampus	0,862	L_hippocampus	0,841	L_hippocampus	0,637	L_hippocampus	0,612	L_hippocampus	0,699	L_hippocampus	0,596
L_pallidum	0,888	L_pallidum	0,781	L_pallidum	0,787	L_pallidum	0,602	L_pallidum	0,667	L_pallidum	0,560
L_putamen	0,857	L_putamen	0,879	L_putamen	0,830	L_putamen	0,638	L_putamen	0,741	L_putamen	0,674
L_thalamus	0,881	L_thalamus	0,784	L_thalamus	0,797	L_thalamus	0,695	L_thalamus	0,651	L_thalamus	0,666
L_ventraldc	0,877	L_ventraldc	0,782	L_ventraldc	0,710	L_ventraldc	0,622	L_ventraldc	0,717	L_ventraldc	0,584
R_accumbens	0,906	R_accumbens	0,926	R_accumbens	0,857	R_accumbens	0,649	R_accumbens	0,870	R_accumbens	0,837
R_amygdala	0,878	R_amygdala	0,868	R_amygdala	0,777	R_amygdala	0,675	R_amygdala	0,727	R_amygdala	0,711
R_caudate	0,823	R_caudate	0,861	R_caudate	0,792	R_caudate	0,640	R_caudate	0,688	R_caudate	0,727
R_hippocampu	0,844	R_hippocampu	0,816	R_hippocampu	0,744	R_hippocampu	0,619	R_hippocampu	0,674	R_hippocampu	0,636
R_pallidum	0,897	R_pallidum	0,846	R_pallidum	0,730	R_pallidum	0,625	R_pallidum	0,703	R_pallidum	0,611
R_putamen	0,864	R_putamen	0,879	R_putamen	0,812	R_putamen	0,678	R_putamen	0,697	R_putamen	0,673
R_thalamus	0,864	R_thalamus	0,804	R_thalamus	0,752	R_thalamus	0,707	R_thalamus	0,695	R_thalamus	0,653
R_ventraldc	0,871	R_ventraldc	0,780	R_ventraldc	0,781	R_ventraldc	0,587	R_ventraldc	0,670	R_ventraldc	0,576
